# PTPN13 induces cell junction stabilization and inhibits mammary tumor invasiveness

**DOI:** 10.7150/thno.38537

**Published:** 2020-01-01

**Authors:** Mohamed Hamyeh, Florence Bernex, Romain M. Larive, Aurélien Naldi, Serge Urbach, Joelle Simony-Lafontaine, Carole Puech, William Bakhache, Jérome Solassol, Peter J. Coopman, Wiljan J.A.J Hendriks, Gilles Freiss

**Affiliations:** 1 IRCM, Institut de Recherche en Cancérologie de Montpellier, INSERM U1194, Université de Montpellier, Institut régional du Cancer de Montpellier, Montpellier, F-34298, France.; 2 RHEM, BioCampus Montpellier, CNRS, INSERM, University of Montpellier, Montpellier, France.; 3 Dynamique des Interactions Membranaires Normales et Pathologiques, CNRS, UMR5235, Montpellier, France.; 4 Institute of Functional Genomics, Montpellier, France; 5 Department of Pathology, CHU Montpellier, Montpellier, France.; 6 Department of Cell Biology, Radboud University Medical Center, Radboud Institute for Molecular Life Sciences, Nijmegen, The Netherlands.

**Keywords:** Breast cancer, Tyrosine Phosphatase, Transgenic mice, metastases, cell junctions, PTPN13.

## Abstract

Clinical data suggest that the protein tyrosine phosphatase PTPN13 exerts an anti-oncogenic effect. Its exact role in tumorigenesis remains, however, unclear due to its negative impact on FAS receptor-induced apoptosis.

**Methods:** We crossed transgenic mice deleted for PTPN13 phosphatase activity with mice that overexpress human HER2 to assess the exact role of PTPN13 in tumor development and aggressiveness. To determine the molecular mechanism underlying the PTPN13 tumor suppressor activity we developed isogenic clones of the aggressive human breast cancer cell line MDA-MB-231 overexpressing either wild type or a catalytically-inactive mutant PTPN13 and subjected these to phosphoproteomic and gene ontology analyses.

We investigated the PTPN13 consequences on cell aggressiveness using wound healing and Boyden chamber assays, on intercellular adhesion using videomicroscopy, cell aggregation assay and immunofluorescence.

**Results:** The development, growth and invasiveness of breast tumors were strongly increased by deletion of the PTPN13 phosphatase activity in transgenic mice. We observed that PTPN13 phosphatase activity is required to inhibit cell motility and invasion in the MDA-MB-231 cell line overexpressing PTPN13. *In vivo*, the negative PTPN13 effect on tumor invasiveness was associated with a mesenchymal-to-epithelial transition phenotype in athymic mice xenografted with PTPN13-overexpressing MDA-MB-231 cells, as well as in HER2-overexpressing mice with wild type PTPN13, compared to HER2-overexpressing mice that lack PTPN13 phosphatase activity. Phosphoproteomic and gene ontology analyses indicated a role of PTPN13 in the regulation of intercellular junction-related proteins. Finally, protein localization studies in MDA-MB-231 cells and HER2-overexpressing mice tumors confirmed that PTPN13 stabilizes intercellular adhesion and promotes desmosome formation.

**Conclusions:** These data provide the first evidence for the negative role of PTPN13 in breast tumor invasiveness and highlight its involvement in cell junction stabilization.

## Introduction

Breast cancer is the most common malignancy in women with about one million new cases per year worldwide. The clinical issue is generally not the primary tumor, which can be surgically removed, but the metastatic spread that is the leading cause of death in patients with breast cancer. One of the first and crucial steps in the metastatic cascade is the acquisition of the invasive capacity. This is accompanied by the reorganization of the actin cytoskeleton related to the disruption of intercellular junctions and loss of cell polarity.

Breast cancer is a heterogeneous disease with three major subtypes 1,2. Two of them are characterized by expression of estrogen receptors [ER] and progesterone receptors [PR]), or overexpression of the oncogenic receptor tyrosine kinase HER2. Therapies targeting these receptors have led to a significant increase in patient survival 3,4. Conversely, the triple-negative breast cancer (TNBC) subtype is defined by the absence of HER2 overexpression and of ER or PR expression, highlighting our lack in understanding the pathways driving TNBC and urging for development of targeted therapies for TNBC as well. Indeed, this breast cancer subtype is very aggressive and associated with poor prognosis 5. TNBC is mostly characterized by the hyper-activation of phosphorylation cascades generally initiated on tyrosine residues. *In vivo*, this reversible and dynamic phosphorylation is initiated by protein tyrosine kinases (PTK). Protein tyrosine phosphatases (PTPs) also regulate tyrosine phosphorylation levels and, in principle, serve as antagonists to PTK signaling 6. However, little is known about PTP role in suppressing tumorigenesis.

The PTP non-receptor type 13 (PTPN13, also called PTPL1, FAP-1, PTP-BAS, PTP1E) is the PTP with the highest molecular weight (270 kDa) 7,8, but its physiological role remains largely unknown. Mice lacking the tyrosine phosphatase activity of PTP-BL (the PTPN13 mouse homolog) show mild impairment of motor nerve repair 9. Our group described PTP-BL role in adipocyte differentiation 10 and reported the first evidence of PTPN13 tumor-suppressive, anti-growth factor effect, via insulin receptor substrate 1 (IRS1) dephosphorylation, in human breast cancer cell lines incubated with anti-estrogens 11-13. Other groups confirmed PTPN13 tumor suppressor properties 6,14-16 and its role in tumor sensitivity to tyrosine kinase inhibitors 17. PTPN13 expression is frequently downregulated or silenced through promoter hypermethylation or loss of heterozygosity in several tumor types 18-20. Moreover, in a large study on colorectal carcinoma, *PTPN13* was identified as one of the three most frequently mutated PTPs and some of these mutations were also found in tumors from other tissues 21. The *PTPN13* gene is located on chromosome 4q21, a region frequently deleted in ovarian, lung and liver cancer 22. In addition, *PTPN13* mRNA expression is an independent prognostic marker of increased overall survival in breast cancer 23, in hepatocellular carcinoma 24, lung cancer 16 and in high grade serous ovarian cancer 25. Finally, we found that *PTPN13* silencing in poorly invasive, hormone-dependent MCF7 breast cancer cells increases the growth of MCF7 cell xenografts in the mammary fat pad of athymic mice, through Src dephosphorylation 13. However, PTPN13 exact role in tumorigenesis remains unclear 8,26, and some findings suggest that it may act as a tumor promoter via inhibition of FAS-induced apoptosis 27,28, or by undefined mechanisms in Ewing's sarcoma 29.

To clarify PTPN13 role in mammary tumorigenesis, we used for the first time genetically-engineered mice. We found that deletion of PTP-BL enzymatic activity in MMTV-HER2 mice accelerates the development and growth of breast tumors and enhances their invasiveness. Furthermore, using hormone-independent MDA-MB-231 cells as a model of human TNBC, we demonstrated that PTPN13 overexpression inhibits cell invasiveness through cell junction stabilization.

## Materials and methods

### Cell lines and antibodies

MDA-MB-231 cells were cultured in DMEM, MCF-7 cells in Ham's F12/DMEM (50%/50%), all supplemented with 10% FBS.

The Flp-In MDA-MB-231 clones that contain a unique Flp recombination target (FRT) site were obtained by stable transfection of pFRTLacZeo (Invitrogen) and selection with zeocin. One clone with a unique FRT site insertion was selected as Mock clone. The Flp-In MDA-MB-231 cells that express wt PTPN13 or the catalytically inactive CS mutant (C 2389 to S) were generated following the manufacturer's instructions. Briefly, HA-tagged PTPN13 and PTPN13 CS 30 were cloned in the pcDNA5/FRT vector (Invitrogen) to generate the pcDNA5/FRT/PTPN13 and pcDNA5/FRT/PTPN13-CS plasmids. pcDNA5/FRT/PTPN13 (and CS) and pOG44 (Invitrogen) were co-transfected at a ratio of 1:9 (w/w) in Flp-In MDA-MB-231 cells and clones resistant to hygromycin B (500 µg/ml) were selected. Expression of wt PTPN13 was confirmed in three selected clones (N13-1, N13-2 and N13-3) and of mutant PTPN13 in one clone (CS).

The following monoclonal and polyclonal antibodies were used: anti-HA (12CA5, Roche), anti-phosphotyrosine (PY99, Santa Cruz Biotechnology), anti-actin (A3854, Sigma), anti-PTPN13 (AF3577, R&D System), anti-E-cadherin (36E, BD Biosciences), anti-desmoglein 2 (Ab150372-Ab151445, Abcam), anti-desmoplakin I+II (Ab16434, Abcam, and DP447-murin, Progen), anti-ERK (9102, Cell Signaling technology/CST) and anti-phosphorylated ERK (P202-204, CST), anti-Src (32G6, CST) and anti-phosphorylated Src (P416, CST), anti-AKT (9272, CST) and anti- phosphorylated AKT (P473, CST). Anti-mouse IgG1 + IgG2a + IgG3 rabbit antibody (ab133469, Abcam) was used as secondary antiserum.

### Animal studies

For xenograft experiments, MDA-MB-231 cells were trypsinized, resuspended in complete medium, counted, pelleted by centrifugation, washed once with ice-cold PBS, pelleted and resuspended (2 × 10^7^ cells per mL) in ice-cold 50:50 solution of Matrigel (Growth Factor-Reduced and Phenol Red-free; #356231, BD Biosciences) and PBS. Fifty microliters of the final cell suspension (10^6^ cells) was injected into the right inguinal mammary gland of anaesthetized 8-week-old female nude mice (8 per group) using a 25-gauge needle. Tumor growth was quantified by measuring the tumor length (*L*), width (*W*) and depth (*D*). These parameters were used to calculate the total tumor volume at the indicated time points using the mathematical formula: tumor volume = *πLWD*/6. Experiments were ended when animals exhibited either large tumors (volume > 1500 mm^3^) or obvious signs of discomfort, or when animals reached the age of 107 days. After sacrifice, liver and lung were frozen in liquid nitrogen and stored at -70°C for PCR analysis; tumors were collected as described in the Histological analysis section.

For liver or lung micro-metastases (DNA level), human DNA (chromosome 17‑specific alpha satellite DNA) was detected as follows. Genomic DNA was isolated from the indicated tissues using Chelex 100 (Sigma) and proteinase K 31. DNA from MDA-MB-231 cells was used as positive control and liver DNA from a control (non-injected) mouse as negative control. Quantitative polymerase chain reactions (qPCR) were performed using a LightCycler 3 apparatus (Roche) with the LightCycler FastStart DNA Master PLUS SYBR Green Kit (Roche), according to the manufacturer's instructions, and the following primers: mouse IL8-F: CTT CGC TGT CGT CCT TGT CT and IL8-R: AGC CAT GAT CCT GAG AAG TCC AT (annealing temperature 62°C); and human chromosome 17‑specific alfa satellite DNA CR 17-1a: GGG ATA ATT TCA GCT GAC TAA ACA G and CR 17 4b: AAA CGT CCA CTT GCA GAT TCT AG (annealing temperature 62°C). The amount of human DNA, which reflects the number of human cancer cells, was determined using the comparative cycle threshold (C_T_) method where C_T_ is defined as the number of cycles at which the fluorescent signal is first detectable. The relative human DNA concentration was obtained by comparing the ΔC_T_ (C_T_ CR-17 - C_T_ IL8) of each sample to the calibration curve obtained using serial dilutions of MDA-MB-231 cell DNA in the control mouse liver DNA.

For transgenic mice experiments, PTP-BL-ΔP mice 9 were backcrossed with wt FVB mice for six generations to introduce the targeted PTPN13 alleles into the FVB background. The wild type PTP-BL protein and the truncated variant PTP-BL-ΔP are expressed at similar levels on this genetic background (Figure S1A). MMTV-HER2 transgenic mice in the FVB strain were generated by Finkle et *al* 32. MMTV-HER2 and PTP-BL- ΔP mice were mated to generate females of the required genotypes: heterozygous for MMTV-HER2 and wt for PTP-BL (HER2+/BL-wt), or heterozygous for MMTV-HER2 and homozygous for PTP-BL ΔP (HER2+/BL-ΔP). Genotypes were determined by PCR analysis as previously described 9,32. Mice were kept on a 12h light/12h dark cycle and were allowed free access to food and water. Animal well-being was monitored daily and tumor occurrence weekly for 57 weeks. Mice carrying tumors >1500 mm^3^ in volume were sacrificed, and tumor, lungs, liver and mammary glands were collected as described in the Histological analysis section.

### Histological analysis

Complete necropsies were performed and tissues used for histology were fixed in 10% neutral buffered formalin and embedded in paraffin. Then, 4 µm-thick sections were cut, stained with hematoxylin and eosin to determine the following histo-pathological characteristics: number of tumor sites per slide; multi-lobulated tumor (grade 1) or not (grade 0); presence of a cystic cavity (grade 1) or not (grade 0) in the nodule; skin ulceration (grade 1) or not (grade 0); tumor necrosis (percentage of necrosis evaluated semi-quantitatively); mitotic index (number of mitoses per field at 40x magnification in 5 fields); angiogenesis (semi-quantitative evaluation of the number of capillaries within the tumor, graded as 1: minimal, 2: slight, 3: moderate); intra-tumor fibrosis (semi-quantitative evaluation of collagen fiber deposition within the tumor, graded as 0: absent, 1: minimal, 2: slight, 3: moderate); invasion of the neighboring environment (semi-quantitative evaluation of EMT at the periphery of tumor islands, graded as 0: absent, 1: minimal, 2: slight, 3: moderate); embolization of tumor cells in a vessel (grade 1) or not (grade 0).

### Western blot analysis

Cells were washed twice in ice-cold PBS and lysed in lysis buffer (40mM Tris-HCl, pH8; 5mM MgCl_2_; 40mM Na_4_P_2_0_7_; 1% Triton X-100, 10mM EDTA, 50mM NaF, 100µM Na_3_VO_4_, 1/250 aprotinin, 1mM AEBSF). Equal protein amounts for each lysate were separated on 7.5 or 10% SDS/polyacrylamide gels before immunoblotting, as previously described (30], with the indicated antibodies.

### Cell growth assay

For cell growth analysis, 1 000 (for day 6 analysis) and 10 000 cells/well (for day 0 analysis) were seeded on 96-well plates. Living cells were quantified at 24h post-seeding (i.e., day 0) and at day 7 post-seeding (i.e., day 6), using the CellTiter 96® Aqueous One Solution Cell Proliferation Assay (Promega). Cell growth was calculated as the signal at day 6 multiplied by 10 and divided by the signal at day 0.

### Wound healing assay

For wound healing experiments, 500,000 cells were seeded in 6-well plates and cell layers (90-100% confluence) were wounded by tip-scraping 24h later. Cells were washed with medium to remove floating cells, and then fresh medium supplemented with 10% FBS was added. Wound healing was imaged at 0 and 9 hours post-wounding with an inverted phase-contrast microscope (Primovert, Zeiss). Wound closure was monitored using a reference point in the wound field. The procedure allowed imaging an identical spot at 0 and 9 hours. The wound width was evaluated at four locations for each scratch using the ImageJ software.

### Transwell invasion assay

Cells were detached with trypsin-EDTA, and resuspended in DMEM supplemented with 1% FBS. 3x10^4^ cells/well were seeded in triplicate in the upper chamber of a BD BioCoat Cell Culture insert (8 µm diameter pore; BD Biosciences) pre-coated with 30 µg of Matrigel (Becton Dickinson). The lower chamber contained DMEM supplemented with 10% FCS as chemoattractant. After 24h, the remaining cells in the upper chamber were scraped off the filter. Cell migration through the filter was evaluated using the colorimetric MTT assay, as previously described 13.

### Cell tracking assay

500 cells per well were seeded in 96-well plates. 24h later, phase-contrast time-lapse images were acquired using the IncuCyte Live Cell Analysis Imaging System (Essen Bioscience) every 15 minutes for 24h. Cells were kept in normal culture conditions in a temperature-controlled incubation chamber and 5% CO_2_ humid air perfusion. Cell tracking analyses were performed using ImageJ and the MTrack2 plug-in (National Institutes of Health, Bethesda, MD) on an average of 250 cells/well followed for at least 12h.

### Quantification of cell-cell adhesion dynamics

Cell-cell interactions were analyzed on time-lapse phase-contrast images collected every 15 minutes for 24h using the IncuCyte Live Cell Analysis Imaging System (Essen Bioscience). Contacting cells during the first 2h of observation were tracked and the duration of cell-cell contact was recorded. Two cells were categorized as remaining in contact, if their cell bodies appeared to be touching upon visual inspection of the image.

### Proteomics

#### Cell culture and sample preparation

For SILAC experiments, a modified method previously described was used 33. Briefly, two opposite biological replicates were performed simultaneously by switching the culture conditions and the cell lines (N13-2 and CS clones). Cells were grown either in heavy or light medium. SILAC media composition: the appropriate amounts of heavy L-arginine [13C6,15N4] and heavy L-lysine [13C6,15N2] (Cambridge Isotope Laboratories) or light L-arginine [12C6,14N4] and light L-lysine [12C6,14N2] (Sigma, Saint Quentin Fallavier, France) were added to lysine- and arginine-free DMEM (Euromedex, Souffelweyersheim, France) supplemented with 10% dialyzed FBS (Invitrogen Corp.) and L-methionine (100 mg/ml; Sigma, Saint Quentin Fallavier, France).

After 11 days of cell culture, cells were serum-starved overnight and then stimulated by switching to complete medium for 30min. The isotope integration rate reached 95%. Cells were lysed as described in the Western blot analysis section. After overnight incubation with anti-phosphotyrosine immunoaffinity beads PY99 (Santa Cruz Biotechnology) at 4°C under rotation, phosphoproteins were then subjected to DTT reduction and IAA alkylation, and size-separated on 12% SDS-PAGE gels. Proteins were in-gel digested with trypsin (GOLD Promega 1 μg/μl in 50mM acetic acid). The resulting peptides were extracted by series of acetonitrile dehydration/triethylammonium bicarbonate buffer (1M) rehydration and vacuum-dried.

#### Mass spectrometric analysis

Samples (1 µl) were analyzed online using a nonoESI Qexactive mass spectrometer (Thermo Fisher Scientific, Waltham, MA) coupled with a RSLC HPLC (Dionex, Amsterdam, The Netherlands). Sample desalting and pre-concentration were performed on-line on a Pepmap® precolumn (0.3 mm x 10 mm). A gradient consisting of 2-24% B for 68min, 24-40% B for 15min, 40-72% B for 5min, and 80% B for 10min (A = 0.1% formic acid, 2% acetonitrile in water; B = 0.1 % formic acid in acetonitrile) at 300 nl/min was used to elute peptides from the capillary (0.075 mm x 150 mm) reverse-phase column (Pepmap®, Dionex). Nano-ESI was performed with a spray voltage of 1.9 kV and a heated capillary temperature of 250°C. Cycles of one full-scan mass spectrum (350-1500 m/z) at a resolution of 70,000, followed by ten data-dependent MS/MS spectra were repeated continuously throughout the nanoLC separation. All MS/MS spectra were recorded at a resolution of 17,500 with an isolation window of 2 m/z (AGC target 1e5, NCE 26). Data were acquired using the Xcalibur software (v 2.2, Thermo Fisher Scientific, Waltham, MA).

Raw data were analyzed using the MaxQuant software (V. 1.4.1.2). Retention time-dependent mass recalibration was applied with the aid of a first search as implemented in the Andromeda software, and peak lists were searched against the UniProt human database (Complete proteome set with isoform; http://www.uniprot.org), 255 frequently observed contaminants as well as reversed sequences of all entries. The standard MaxQuant settings were used. Enzyme specificity was set to Trypsin/P. Up to two missed cleavages were allowed and only peptides with at least seven amino acids in length were considered. Methionine oxidation and serine, threonine and tyrosine phosphorylation were set as variable modifications. Peptide identifications were accepted based on their false discovery rate (FDR, 1%). Accepted peptide sequences were subsequently assembled by MaxQuant into proteins, to achieve a false discovery rate of 1% at the protein level. Relative protein quantification in samples to be compared was performed based on the median SILAC ratios, using the MaxQuant software with standard settings.

#### Statistical analysis

Proteins quantified in both biological replicates with at least a total number of three peptide evidences were selected. To analyze the normalized SILAC ratios of all peptide evidences of each protein, an empirical estimation of their FDR was performed based on the non-parametric Wilcoxon rank test. Proteins were accepted as differentially enriched based on their FDR (0.05%) and their SILAC ratio (>20% of variation in both biological replicates). Details are in Supplementary Methods.

### Cell aggregation assay

Cell aggregation assays were performed as described by Boterberg *et al* 2001 34. Briefly, cells were washed with Mg/Ca-free PBS and then completely dissociated by trypsinization at 37°C for 2min. Then, 150,000 cells were seeded in triplicate in 24-well ultra-low attachment plates (Corning) with 4mM CaCl_2_ or 1mM EGTA chelator. Plates were rotated at 80 rpm on a POS-300 Grant-bio rotator in a cell culture incubator for 18h. The formed aggregates were carefully spread over the well by pipetting, fixed with 2% PFA for 20min, and then stained with Hoechst. The aggregate size and number were measured using Cellomics BioApplications (Thermo Scientific) with a Zeiss 20X 0,4 NA Korr LD Plan Neofluar lens.

### Quantitative RT-PCR

RNA was isolated using the Tri Reagent (Zymo Research) according to the manufacturer's instructions, and quantified by measuring the absorbance at 260 nm. RNA quality was checked by assessing the ratio between the absorbance values at 260 and 280 nm. Total RNA (1 μg) was reverse-transcribed using 5 μM of random hexamers (Roche) and Superscript II, according to the manufacturer's instructions (Invitrogen). Quantitative real-time polymerase chain reaction (qPCR) analysis was then performed using the amount of cDNA corresponding to 12.5 ng of total RNA on a Light Cycler 3 apparatus (Roche) with the Light Cycler FastStart DNA Master PLUS SYBR Green I Kit (Roche), according to the manufacturer's instructions. The primers used for PCR amplification were: PTPN13 forward 5' CAG TCA CAG AGA CCG AGC AGA CAA 3', reverse 5' TGC CGT TTT AGC ATG ATC TCT TGA 3'; vimentin forward 5' GAG AAC TTT GCC GTT GAA GC 3', reverse 5' gct tcc tgt agg tgg caa tc 3'; Zeb-1 forward 5' gtt ctg cca aca gtt ggt tt 3', reverse 5' gct caa gac tgt agt tga tg 3'; SLUG forward 5' cag tgg ctc aga aag ccc c 3', reverse 5' tca gct tca atg gca tgg g 3'; E-cadherin forward 5' tgc cca gaa aat gaa aaa gg 3', reverse 5' gtg tat gtg gca atg cgt tc 3'; SNAIL forward 5' gct gca gga ctc taa tcc aga 3', reverse 5' gac aga gtc cca gat gag cat 3'; HPRT forward 5' CTG ACC TGC TGG ATT ACA 3', reverse 5' GCG ACC TTG ACC ATC TTT 3'. The relative quantification of each candidate gene expression was performed with the comparative cycle threshold (C_T_) method using Mock cells as the calibrator sample (expression set at 1), and HPRT values as endogenous RNA normalization control 35.

### Immunofluorescence analysis

Cells plated on coverslips were fixed with 3.7% formaldehyde in PBS at room temperature for 15min (for desmoglein immunodetection) or in methanol at -20°C for 15min (for desmoplakin immunodetection), and permeabilized with 0.5% Triton X-100 in PBS for 10min. Immunolabeling was performed as described 30 with FITC-conjugated secondary antibodies (Jackson ImmunoResearch Laboratories). Coverslips were mounted and images were taken on a Zeiss Imager M2 using a PlanApochromat 63x /1.3 DIC (oil) objective (Nikon). Tumor tissues were fixed in 10% neutral buffered formalin and embedded in paraffin, sectioned (4 µm-thick), deparaffinized, permeabilized with 0.1% Tween-20 in PBS and blocked with 3% BSA. Immunolabelling was performed using undiluted anti-desmoplakin (DP447, Progen) antibody for 12h, followed by the anti-mouse IgG1 + IgG2a + IgG3 rabbit antibody (ab133469, Abcam) (1/2000) for 8min, and then Alexa Fluor 555-conjugated anti-rabbit secondary antibodies (Invitrogen). Coverslips were mounted and images were taken using a Zeiss Imager M2 microscope and a PlanApochromat 40x /1.3 DIC (oil) objective (Nikon) with apotome.

### Patient survival and KM Plotter data

Survival analysis was performed using a breast cancer meta-data set composed of 3,951 samples and the KM-Plotter online analysis tool (www.kmplot.com). The Kaplan-Meier curves of relapse-free survival time, the hazard ratio with 95% confidence intervals and log-rank test P values for 17 years of follow-up were calculated using the 204201_s_at probe set and automatically selected cut-offs.

### Statistical analysis

The specific details of the used statistical tests and number of samples and experimental repeats are included in the figure legends. The Student's *t*-test was used for comparing two groups. The log-rank (Mantel-Cox) test was used for comparing survival curves. Two-way ANOVA was used for comparing tumor growth curves. All tests were two-sided with α level = 0.05 and all tests were performed with Prism (GraphPad software). *P* values lower than 0.05 were considered significant.

## Results

### PTPN13 expression is a survival prognostic marker in breast cancer

**Figure 1:**
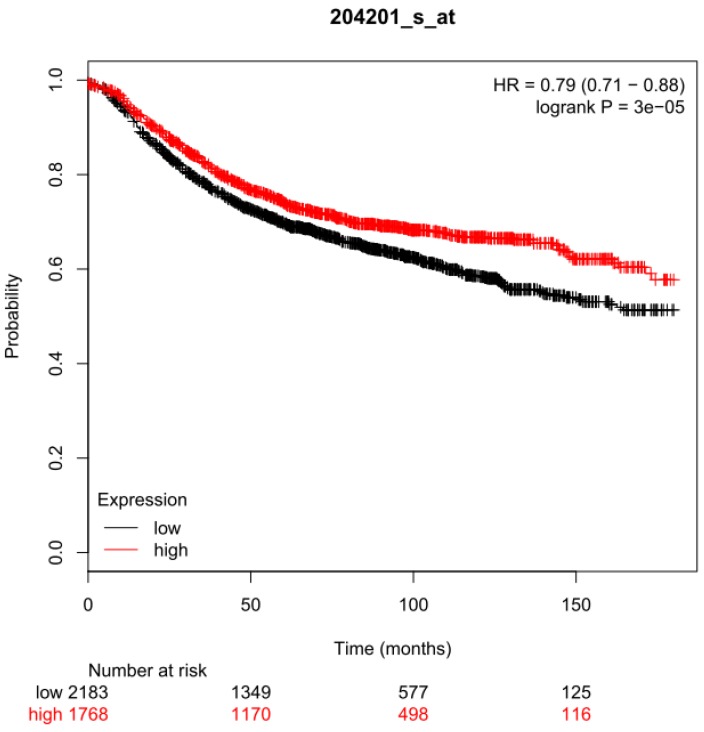
** Prognostic value of PTPN13 expression.** Association of PTPN13 RNA expression with relapse-free survival of breast cancer patients using KM Plotter data (see Methods for analysis details). HR: hazard ratio; log-rank P: P value for curve comparison calculated with the log-rank test. Numbers below the graph indicate the number of patients at risk (total and at the indicated time points); n = 3,951 patients in the KM Plotter meta-data set.

In a previous retrospective analysis of breast tumor RNA samples from 291 patients we showed that *PTPN13* mRNA expression is an independent prognostic marker of increased overall survival 23. We failed, however, to show significant prognostic value for recurrence-free survival due to the small number of samples although this parameter was more closely related to tumor aggressiveness 23. Here, using the KM Plotter data for 3951 breast cancer samples, we confirmed PTPN13 prognostic interest by showing that the recurrence-free survival probability was significantly higher in patients with breast cancer displaying high *PTPN13* expression level compared with those with low expression level (hazard ratio = 0.79) 36 (Figure 1). The confirmation of the prognostic value of PTPN13 justifies determining its role in tumor progression and the underlying mechanisms.

### Breast cancer development is accelerated and tumor invasiveness increased in HER2-overexpressing mice that lack PTPN13 catalytic domain

**Figure 2:**
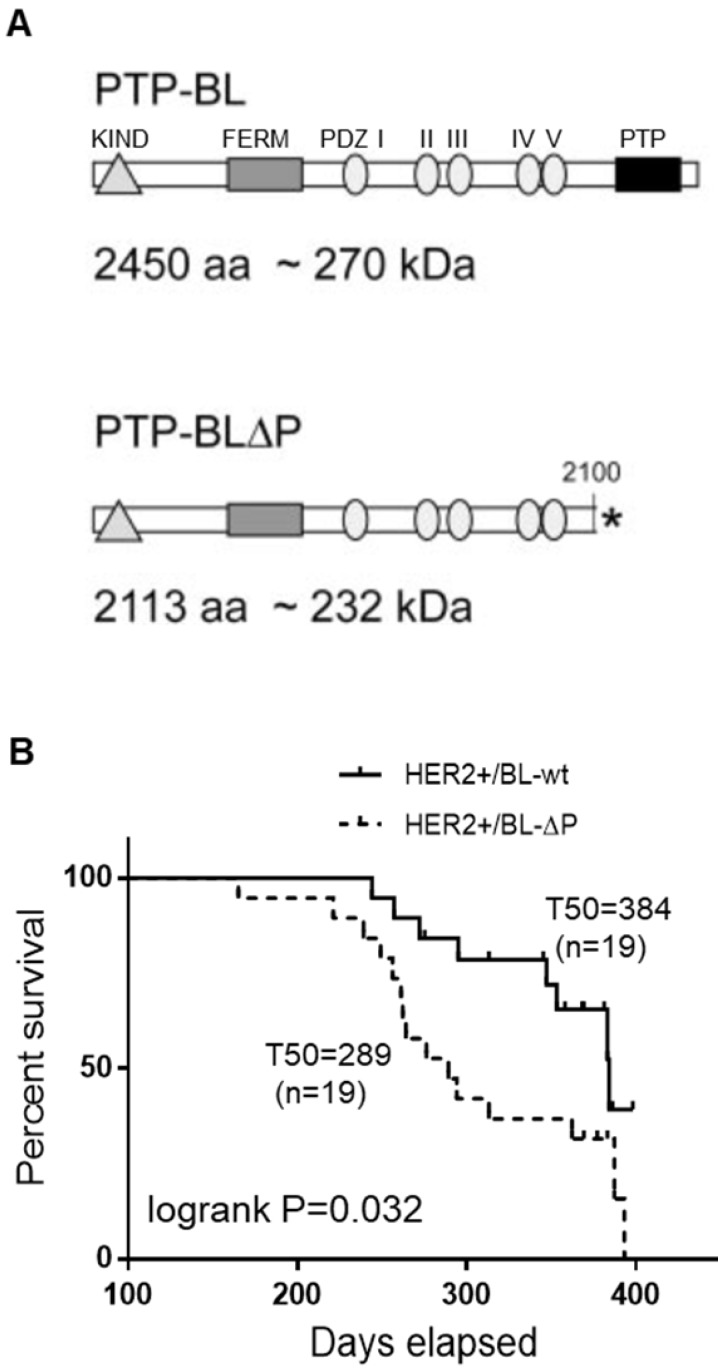
** Lack of catalytically active PTPN13 increases mammary tumor frequency in HER2+ mice. A:** Protein domain structure of PTP-BL and PTP-BL△P. **B:** Kaplan-Meier analysis of tumor occurrence in MMTV-HER2; PTP-BL+/+ (HER2+/BL-wt) and MMTV-HER2; PTP-BL△P/△P (HER2+/BL-ΔP) transgenic female mice. The curves were drawn and analyzed using the Prism software. P value obtained using the log rank test. The number of animals analyzed for each genotype (n) and the median time to tumor onset (T50) are also shown.

To explore PTPN13 role in breast tumor development in a relevant genetically-modified mouse model, we crossed mice that lack PTPN13 catalytic domain (PTP-BL-ΔP) or not (PTP-BL-wt) 9 with MMTV-HER2 transgenic mice in which 70% of females develop mammary carcinoma before one year of age 32. Weekly monitoring of tumor development for 57 weeks indicated that tumors appeared between 244 and 384 days in 42% of HER2+/BL-wt, and between 165 and 393 days in 79% of HER2+/BL-ΔP females. Compared with HER2+/BL-wt mice, the median time to tumor onset (T_50_) was significantly shorter in HER2+/BL-ΔP mice (384 days and 289 days, respectively) (Figure 2). Tumor development occurred approximately 100 days earlier and tumor incidence was nearly doubled in mice lacking PTP-BL enzymatic activity. HER2 was shown to be dephosphorylated by PTPN13 in a human breast cancer cell line 37. We did not observe evidence for such a direct effect of PTPN13 on HER2 phosphorylation in tumors expressing PTP-BL-WT versus PTP-BL-ΔP; HER2 is phosphorylated heterogeneously, with a stronger phosphorylation at the edges, in both types of tumors (Figure S1B).

**Figure 3:**
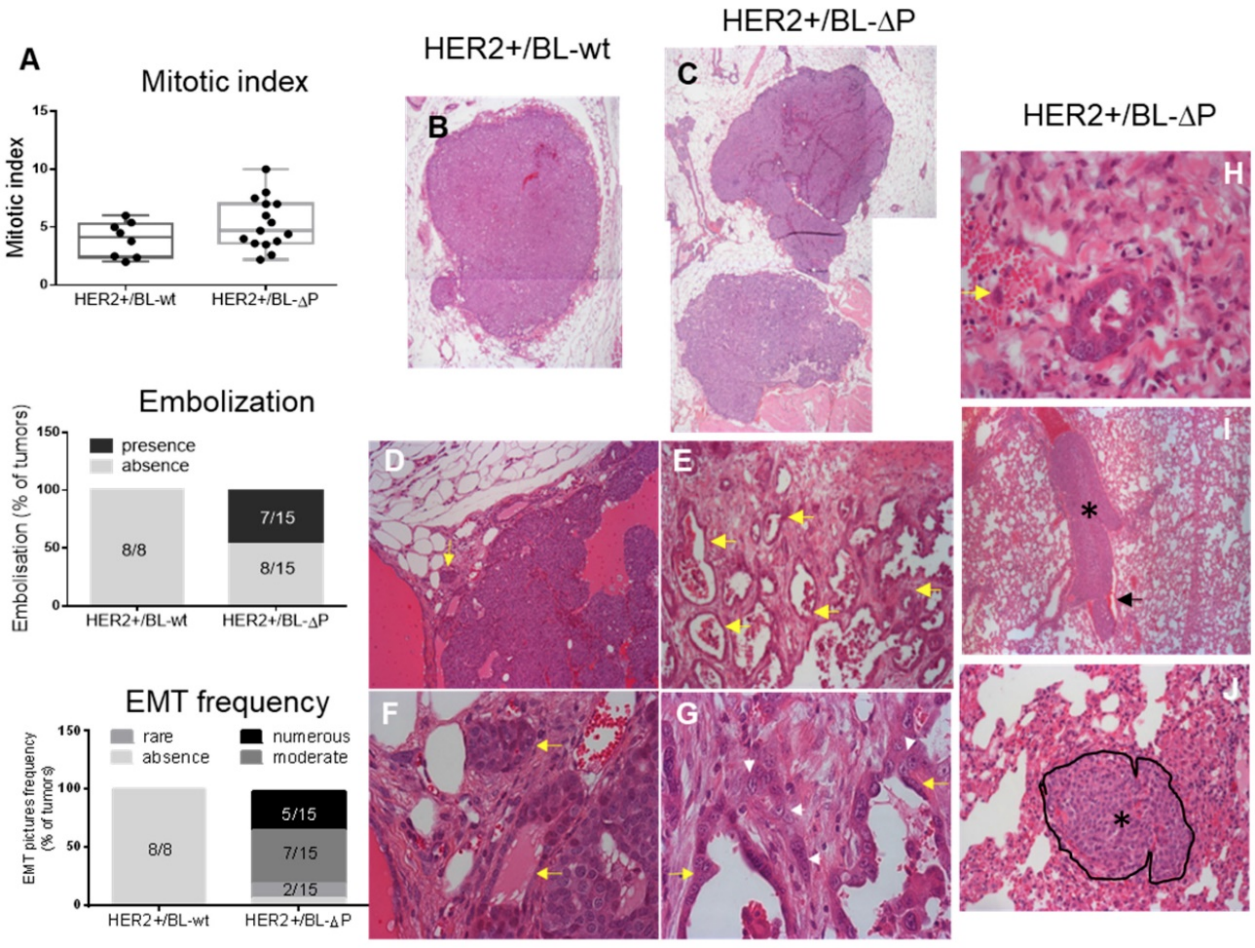
** Lack of catalytically active PTPN13 promotes mammary tumor invasiveness. A:** Mitotic index (box-plot whiskers represent the minimum and maximum values), embolization frequency, and presence of EMT in tumors from MMTV-HER2;PTP-BL+/+ (HER2+/BL-wt) (n=8; panels **B, D, F**) and MMTV-HER2; PTP-BL△P/△P (HER2+/BL-ΔP) (n=15; panels **C, E, G**) mice. **B&C:** Examples of nodules constituting the tumor. Magnification: 2.5x. **D&E:** Examples of the local invasiveness into the surrounding tumor microenvironment. Magnification: 10x. Yellow arrows show cellular invasion in the stroma, with cells arranged in tubules. These events are rare in HER2+/BL-wt and frequent in HER2+/BL-ΔP tumors. **F&G:** EMT is increased in the absence of PTP-BL activity (magnification: x40). The white arrowheads show tumor cells undergoing EMT; yellow arrows show the tubules. **H:** Example of tumor cell embolization in a vein (yellow arrow indicates two tumor cells) from a HER2+/BL-ΔP tumor. At the periphery of the tumor nodule, malignant epithelial cells are infiltrating the microenvironment. Some cells enter vessels. **I:** Lung section from a HER2+/BL-ΔP mouse with massive embolization of tumor cells (black asterisk) in a large lung blood vessel. Erythrocytes (black arrow) are found only at the periphery of tumor cells. Magnification: x5. **J:** Metastatic tumor nodule in the lung of a HER2+/BL-ΔP mouse. The nodule, delineated and indicated by a black asterisk, is located between the alveolar spaces, compresses the surrounding tissue, and infiltrates the stroma. Magnification: x20.

Although the number of mammary nodules was similar in tumors from both strains, lack of catalytically active PTPN13 promotes number of tumor localizations per mouse (Figure S2). Microscopic analysis showed that the histopathology of HER2+/BL-ΔP tumors (n=15) was markedly different from that of HER2+/BL-wt tumors (n=8) (Figure 3A-J): the proportion of multilobular nodules was increased from 42% to 90% (Figure 3B,C); signs of minimal to moderate epithelial-mesenchymal transition (EMT; based on the cell morphology) were present in 93% of HER2+/BL-ΔP tumors, but absent in HER2+/BL-wt tumors (Figure 3A,D,E,F,G); and cancer cell embolization in vessels was detected in 46% of HER2+/BL-ΔP tumors, but absent in HER2+/BL-wt tumors (Figure 3A,H). Conversely, the overall mitotic index was not significantly different between tumor groups (Figure 3A). Finally, microscopic analysis of one lung section of each tumor-bearing mice highlighted massive embolization of tumor cells in a large lung blood vessel in one HER2+/BL-ΔP mouse, and a micro-metastasis in another, but not in HER2+/BL-wt mice (Figure 3I,J).

These results demonstrate that lack of PTPN13 phosphatase activity in MMTV-HER2 mice reduces tumor latency, increases tumor frequency, and also promotes tumor invasiveness and aggressiveness.

### PTPN13 inhibits breast cancer cell motility and invasiveness

**Figure 4:**
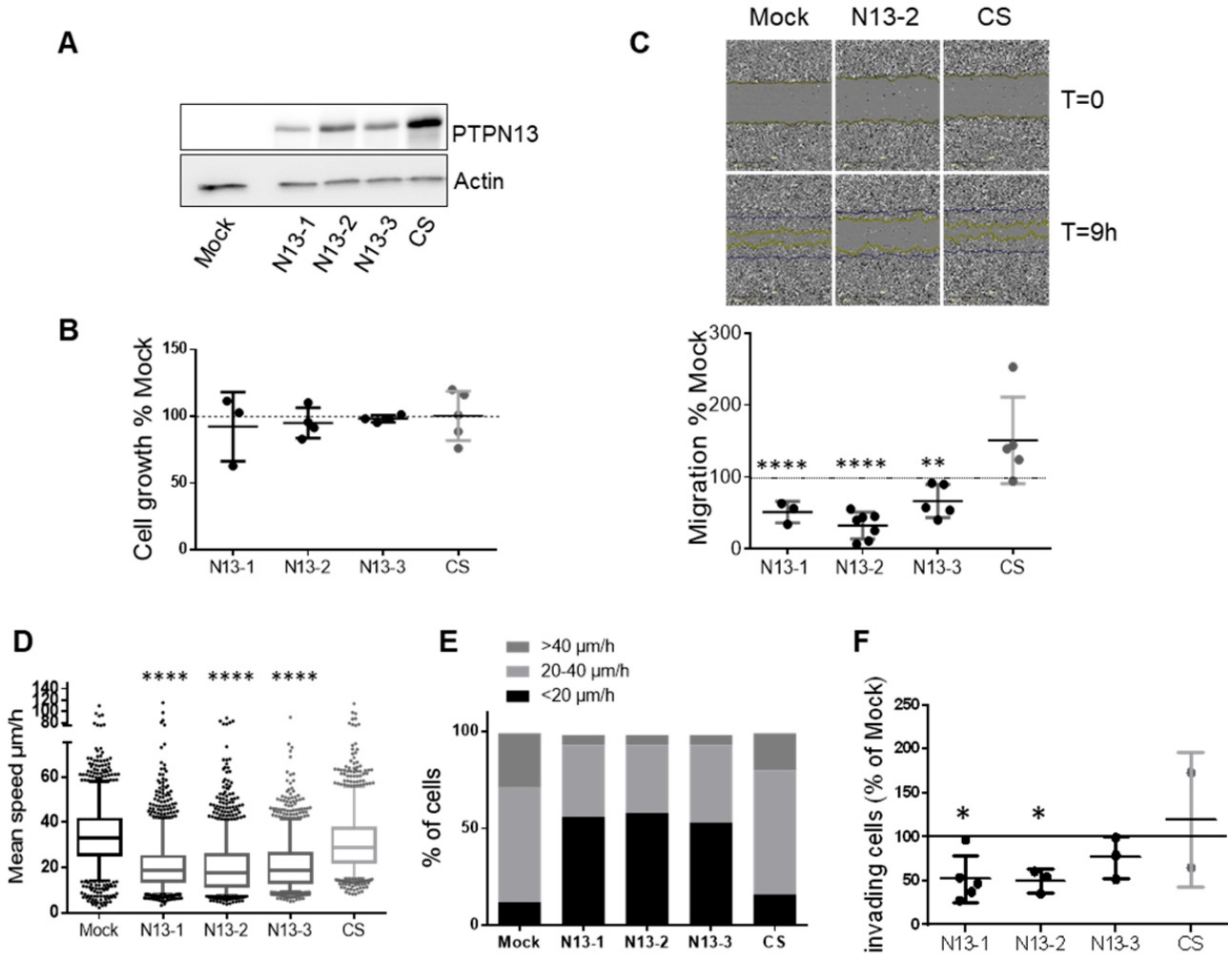
** PTPN13 regulates MDA-MB-231 cell motility and invasiveness. A:** Expression of wt (N13-1, N13-2, N13-3) or catalytically inactive (CS) PTPN13 in the indicated cell clones was monitored by western blotting using anti-PTPN13 antibodies. Mock: control cells (vector alone); equal loading was verified by re-probing with an anti-actin antibody. **B:** Cell growth measured using the MTS assay. Results, expressed as % of Mock cells, are the mean ± s.d. of three (N13-1) four (N13-2, N13-3) or five (Mock, CS) independent experiments. **C:** Directional migration was assessed with the wound healing assay. **C. Upper panel:** Phase-contrast optical photomicrographs of the wounded area at 0 and 9h. **C. Lower panel:** Quantification of cell migration, expressed as % of Mock cells; mean ± s.d. of 3 (N13-1) or ≥5 (Mock, N13-2, N13-3, CS) independent experiments. **P<0.01, ****P<0.0001 versus Mock. **D:** Individual migration of the indicated cell clones was monitored by video microscopy and cell tracking on duplicate wells in three independent experiments. Graph represents the speed of about 1500 cells for each clone, Box-plot whiskers represent the 5 and 95 percentile values; ****P<0.0001 versus Mock and CS. **E:** Classification of cells (percentage) according to their migration speed (as in panel E): slow (<20µm/h), medium (20 to 40µm/h) and fast (>40µm/h). **F:** Cell invasiveness was evaluated with the Boyden chamber test. The percentage of cells that migrated through Matrigel-coated filters was quantified relative to the total number of seeded cells. Results, expressed as % of Mock cells, are the mean ± s.d. of five (N13-1), three (N13-2, N13-3) or two (CS) independent experiments *P<0.05 versus Mock. (**C, D** and** F**) two-tailed Student's t-test.

To extend our knowledge on PTPN13-mediated tumor invasion regulation specifically in TNBC, we generated isogenic MDA-MB-231 cell clones that overexpress wild type (wt) PTPN13 (three clones: N13-1, -2 and -3) or a catalytically inactive mutant (one clone: CS) using the Flp-In system. We chose MDA-MB-231 breast cancer cells because of their very low endogenous PTPN13 levels, high invasive capacity and hormone-independency. All N13 clones expressed comparable PTPN13 protein levels, and the CS clone displayed a slightly higher level (Figure 4A). Expression of wt or mutant PTPN13 did not modify cell growth or apoptosis compared with control (pFRTLacZeo vector alone; Mock) (Figure 4B and S3), but significantly affected cell motility and invasiveness (i.e., two biological parameters associated with tumor cell aggressiveness) (Figure 4C-F). Analysis of the global cell motility with the wound healing assay showed that expression of wt PTPN13 (N13-1, N13-2 and N13-3) reduced migration by 48%, 67% and 46%, respectively, compared with control (Mock) (Figure 4C). Conversely, expression of mutant PTPN13 (CS) slightly increased migration (not significant), suggesting a possible dominant-negative effect.

Quantification of the video-monitoring data of cells seeded at low density showed that in the N13 clones, the migration speed of individual cells was reduced by 35-40% (Figure 4D), and that 4 to 5 times more cells had a speed lower than 20 µm/h (Figure 4E) compared with Mock and CS cells. Altogether these results demonstrate that PTPN13 inhibits breast cancer cell migration through its phosphatase activity.

Finally, invasion assays using Matrigel-coated Boyden chambers showed that invasiveness of N13 cells was reduced by 40-50% compared with Mock and also CS cells (Figure 4F).

These findings indicate that PTPN13 inhibits cell aggressiveness in hormone-independent breast cancer cells.

### PTPN13 phosphatase activity delays orthotopic MDA-MB-231 cell xenograft growth and invasiveness

**Figure 5:**
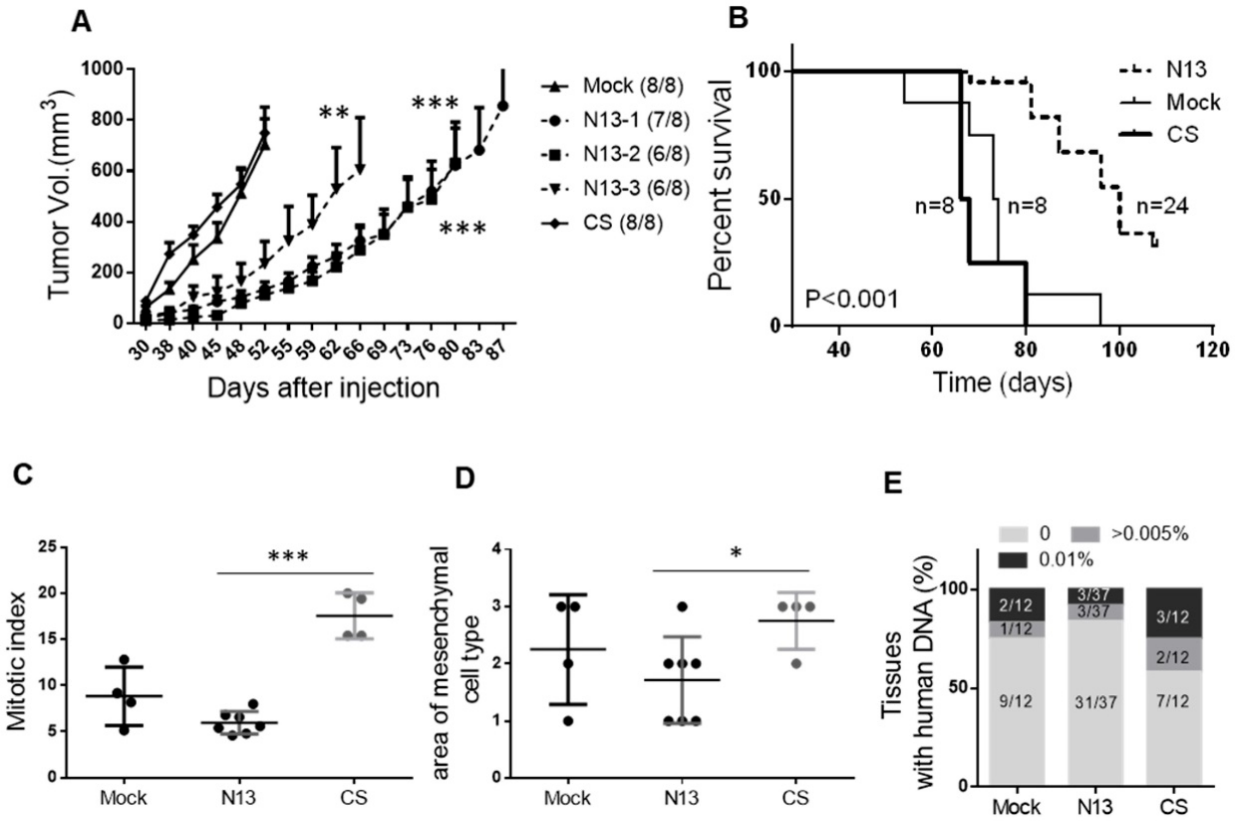
** PTPN13 phosphatase activity delays MDA-MB-231 breast cancer cell xenograft growth in the mammary fat pad and invasiveness.** 10^6^ N13-1, N13-2, N13-3, CS, or control (Mock) cells were injected in the mammary fat pad of 8-week-old female immunodeficient mice. **A:** Tumor growth was measured twice/week and mice were sacrificed when tumor reached 1500mm3. The curve shows the tumor size (mean ± s.e.m.) over time in mice that developed a tumor within 90 days post-injection (numbers in brackets); the curve stops when the first mouse in each group is sacrificed. *** P<0.001, **P<0.01 versus Mock (ANOVA from day 30 to day 52) **B:** Kaplan-Meier analysis of survival (event: tumors reaching 1500mm3). The curves were drawn and analyzed using the Prism software. P value obtained using the log rank test. The number of animals in each group (n) is shown. Mitotic index (**C**) and area of tumor mesenchymal cell type (**D**), expressed as the mean ± s.d., in Mock and CS cell-derived (n=4) and N13 cell-derived tumors (n=7), *** P<0.001, *P<0.05 (two-tailed Student's t-test). **E:** Micro-metastasis detection by PCR. Data represent the percentage of mouse tissue (lung and liver) DNA samples containing also human DNA. The number of tissues tested is shown in brackets.

To validate these *in vitro* findings in an *in vivo* setting, we injected N13-1, N13-2, N13-3, CS or mock MDA-MB-231 cells in the mammary fat pad of female immunodeficient mice (n=8/group) (Figure 5). All mice xenografted with Mock or CS cells developed tumors and were sacrificed before 96 days (tumor volume >1500mm^3^). Conversely, only 19 of the 24 mice injected with N13 cells developed tumors, among which only 15 reached 1500mm^3^ before 107 days (Figure 5B). Mock and CS MDA-MB-231 cell xenografts grew steadily, reaching a mean volume of 512 ± 92 mm^3^ and 547 ± 64 mm^3^ (mean ± s.e.m.), respectively, at day 48 post-injection. At this time-point, the volumes of N-13 cell xenografts were 121 ± 27, 88 ± 23 and 179 ± 74 mm^3^ for N13-1, N13-2 and N13-3, respectively (p=0.0015, 0.0017 and 0.016, respectively, compared with Mock) (Figure 5A). Moreover, the median time before sacrifice (T_50_) was 73 days and 67 days for the Mock and CS groups, respectively, and 100 days for the N13 groups (Figure 5B). Altogether these results demonstrate that PTPN13 can inhibit tumor development and growth through its phosphatase activity.

Microscopic analysis of the tumors at sacrifice showed that the mitotic index was significantly lower in N13 cell-derived tumors (5.9 ± 0.5) than in CS (17.5 ± 1.3) and Mock MDA-MB-231 cell-derived tumors (8.9 ± 1.5; Figure 5C). Furthermore, the number of mesenchymal tumor cell regions was significantly reduced in N13 cell-derived tumors (1.7 ± 0.3) compared with CS (2.75 ± 0.25) and Mock (2.25 ± 0.4) MDA-MB-231 cell-derived tumors (Figure 5D). This suggests that PTPN13 over-expression leads to a mesenchymal-epithelial transition (MET)-like pattern in the MDA-MB-231-derived tumors. Conversely, loss of PTP-BL phosphatase activity in the HER2 mouse model led to an EMT-like pattern in the resulting tumors (see Figure 3G).

We did not observe any macro-metastasis in the lungs and liver of xenografted mice. However, using the PCR technique described by Becker *et al* 38 we detected micro-metastases (i.e., human DNA) in 41% of the liver and lung samples from mice xenografted with CS cells, in 30% of samples from mice xenografted with Mock MDA-MB-231 cells, and only in 18% of samples from mice xenografted with N13 cells (Figure 5E, Figure S4). Collectively, these data indicate that PTPN13 inhibits tumor aggressiveness and that its catalytically-inactive mutant may act as a dominant-negative variant that promotes tumor aggressiveness. This is in agreement, with the phenotype of HER2+/BL-ΔP mice (Figures 3 and 4).

### Phosphoproteomic comparison of N13 and CS MDA-MB-231 breast cancer cells suggests a PTPN13 role in intercellular junctions

**Figure 6:**
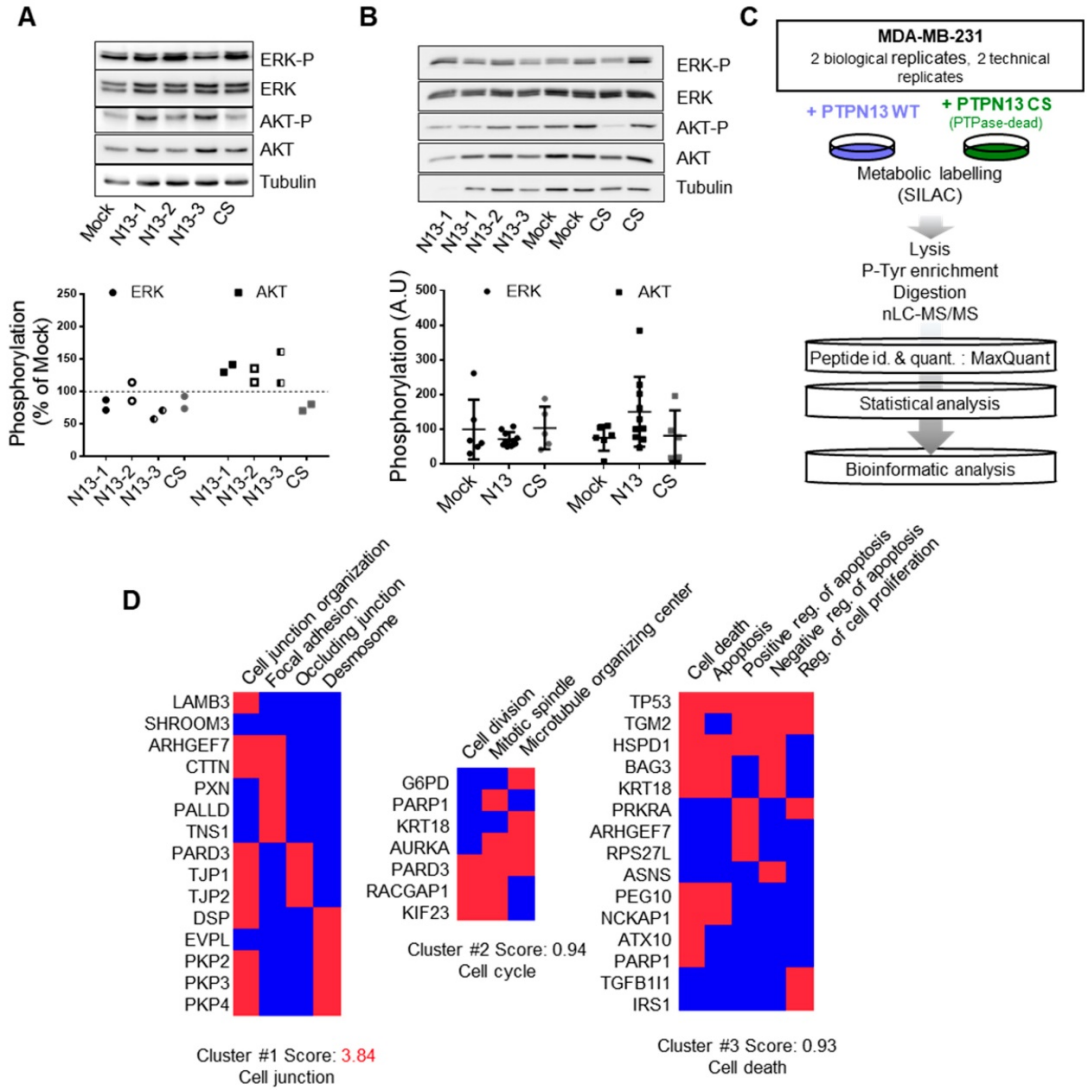
** Phosphoproteomic data elucidate the biological processes affected by PTPN13 in breast cancer cells. A:** AKT and ERK expression and phosphorylation in the indicated clones was assessed by western blotting. Tubulin was used as loading control. Left panel: representative western blot; right panel quantification of phosphorylation relative to that in Mock cells in two independent experiments. **B:** AKT and ERK expression and phosphorylation in the indicated tumor xenograft samples was assessed by western blotting. Tubulin was used as loading control. Left panel: representative western blot; right panel: mean (arbitrary units) ± s.d. of the densitometric analysis of 5 Mock, 5 CS and 10 N13 xenografts. **C:** Experimental design of the SILAC-based quantitative phosphoproteomic analysis. Phosphotyrosine-dependent protein complexes from MDA-MB-231 cells expressing wt PTPN13 or the phosphatase-dead (CS) mutant were identified and quantified by mass spectrometry. To select the 97 PTPN13 targets amongst the 1225 quantified proteins, the SILAC ratios of the peptides assigned to the proteins were evaluated with the Wilcoxon test. Then, after the empirical estimation of the false discovery rate, proteins with a threshold of 0.0005 and more than 20% of variation of SILAC ratios were retained. WT, wild-type; PTPase-dead, phosphatase-dead; id., identification; quant., quantification. **D:** Integrative bioinformatics analysis of the proteins selected as PTPN13 targets. The significantly altered phosphotyrosine-dependent proteins were classified according to their associated GO terms and clustered with the DAVID functional annotation tool. Red boxes: proteins associated with the indicated GO term.

To identify the intracellular signaling pathways implicated in PTPN13 inhibitory effects on MDA-MB-231 cell aggressiveness, we first tested the phosphorylation/expression levels of ERK (involved in cell growth regulation) and of Src and AKT, in view of our previous findings 13,30. In Mock, N13 and CS cell lysates as well as in xenograft tumor lysates, we could not detect any Src activation.* In vitro*, PTPN13 expression slightly increased AKT phosphorylation (Figure 6A) but we did not observe such a correlation *in vivo* (Figure 6B). Similarly, phosphorylation of ERK was not significantly different in cells and tumors expressing wt or mutant PTPN13 compared with Mock (Figure 6A-B).Thus, no indications were obtained that PTPN13's tumor suppressor activity is associated with ERK, AKT and Src inactivation in this model.

We then performed mass spectrometry-based proteomics to compare tyrosine phosphorylation in N13 and CS MDA-MB-231 cells. We used SILAC to label lysine and arginine residues of newly synthesized proteins with different isotopes in N13 and CS cell cultures, and carried out two opposite experiments simultaneously by reversing the cell culture conditions (Figure 6C). The mean labeling efficiency of cells was ≥95% after 11 days in culture. Using the MaxQuant algorithms, which also calculated the exact heavy/light isotope (H/L) ratio of each protein 39, we could identify nearly 1400 different proteins in anti-phosphotyrosine immunoprecipitates obtained from mixed N13 and CS MDA-MB-231 cell extracts (Table S1). The presence of IRS1 and TRIP6, two known PTPN13 substrates 30,40, among the hypo-phosphorylated proteins in N13 as compared to CS samples validated our approach. We selected a list of 97 proteins for further analysis, based on their significantly different H/L ratio and on their inverted H/L ratio >1.2 in the two labeling conditions (Table S2).

The Panther gene ontology WEB tool 41, using the Bonferroni correction, revealed the significant enrichment of differentially tyrosine-phosphorylated proteins that belonged to the “cell junction assembly” and “cell-cell junction organization” biological processes, and to the “desmosome”, “focal adhesion” and ”spindle midzone” cellular component groups. Cluster analysis using the DAVID WEB tool 42,43 highlighted three clusters that corresponded to “cell junction”, “cell division” and “apoptosis” (Figure 6D). Indeed, PTPN13 has been associated with the regulation of cell growth and apoptosis 26, and accumulates at the midbody during cell division 44. However, in the MDA-MB-231 clones used for the proteomic analyses, we did not observe any change in cell proliferation *in vitro* and only a weak effect on tumor mitotic index *in vivo*.

Because the “cell junction” gene cluster ranked top in this analyses, and the “cell junction assembly” and “desmosome” terms were highest in the ontology enrichment analyses, we focused on a cluster of 15 genes that comprise the subheadings “desmosome”, “occluding junction” and “focal adhesion”. We found that all proteins related to focal adhesions were hypo-phosphorylated in N13 cells, while most of the cell-cell junction proteins were hyper-phosphorylated in N13 cells. This suggests a direct or indirect effect of PTPN13 on focal adhesion through phosphorylation/dephosphorylation cascades. The effect on cell-cell junctions, which are absent in parental MDA-MB-231 cells, might be indirect through stabilization, induction of expression, or changes in protein interaction.

### PTPN13 stabilizes intercellular junctions

**Figure 7:**
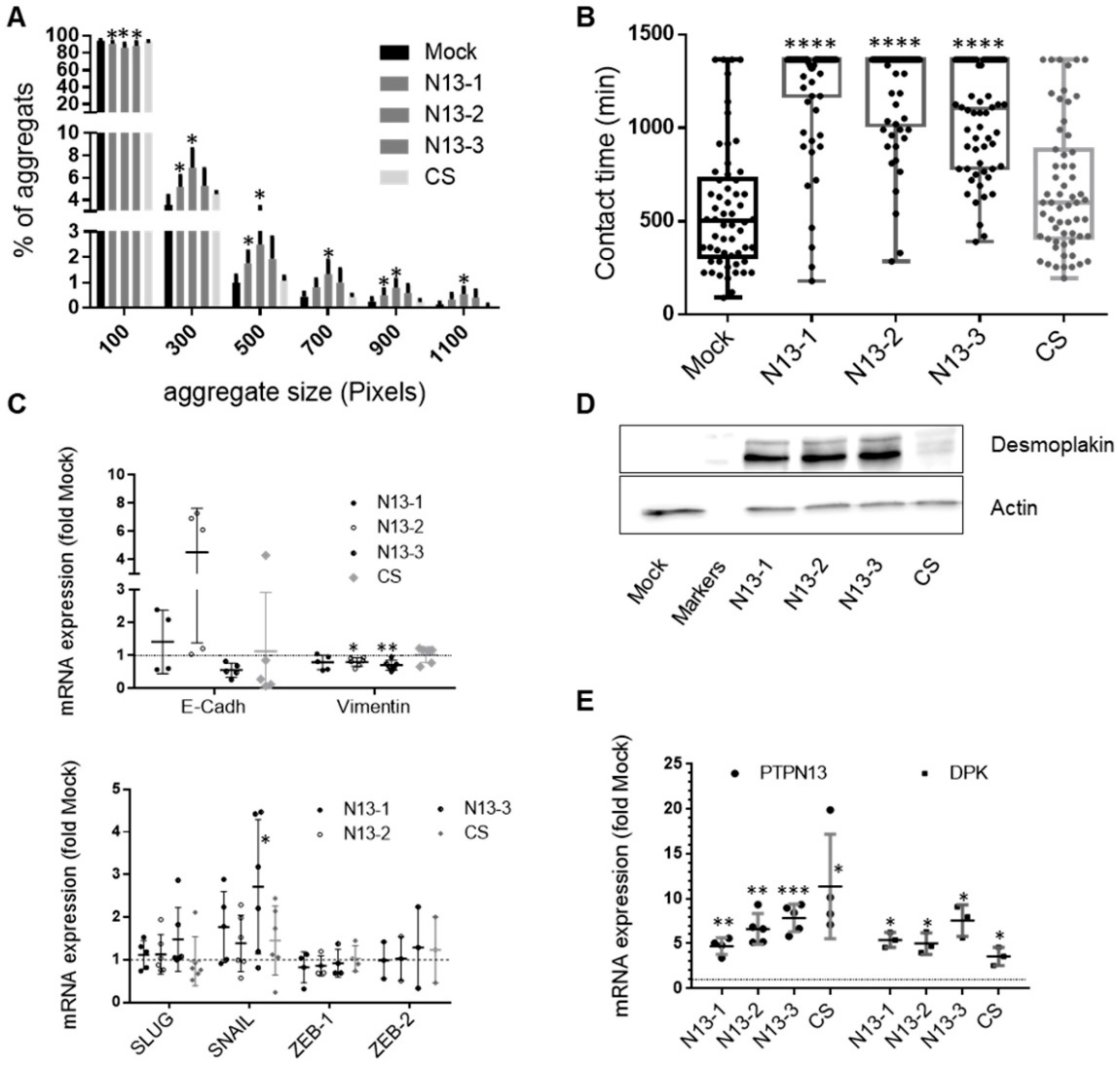
** PTPN13 stabilizes intercellular adhesion. A:** Cell-cell adhesion was evaluated in MDA-MB-231-derived clones using a 3D cell aggregation assay. The percentage of each aggregate size class was calculated and plotted using the Prism software. A 100 pixel surface corresponds approximately to three cells. Results are the mean ± s.d. of four independent experiments. *P<0.05 versus Mock. **B:** Cell-cell adhesion stability was evaluated by video monitoring. The mean contact time between cells (in minutes) was calculated, and values (n>55) were plotted. Box-plot whiskers represent the minimum and maximum values; ****P<0.0001 versus Mock. Note that in N13 cultures, many cells remained in contact during the whole experiment, explaining the lack of upper whiskers and mean values for these clones. **C:** Expression of EMT master genes evaluated by RT-qPCR. Data were normalized to HPRT levels and are the mean ± s.d. mRNA levels relative to those in Mock cells (n≥5 experiments for SLUG, SNAIL and vimentin; n≥4 for E-cadherin and ZEB-1, and n= 3 for ZEB-2). *P<0.05, **P<0.01 versus Mock. **D:** Desmoplakin expression in the indicated cell clones was monitored by western blotting with actin as loading control. **E:** PTPN13 and desmoplakin expression evaluated by RT-qPCR and normalized to that of HPRT. Data are expressed relative to Mock cells, and are the mean ± s.d. (n≥3 for DPK, n≥4 for PTPN13. *P<0.05, **P<0.01, ***P<0.001 versus Mock. (A, B, C and E) two-tailed Student's t-test.

To better understand PTPN13 role in cell-cell interactions, we first assessed cell-cell adhesion in N13, CS and Mock MDA-MB-231 cells in 3D culture conditions using a previously described cell aggregation protocol 45. Cell aggregation was significantly increased in N13 cells; with 2-3-fold more aggregates that contained more than five cells (Figure 7A). To quantify PTPN13 effect on cell-cell interaction dynamics, we identified cell-cell contacts in time-lapse videos and quantified the duration of the intercellular contacts. The mean lifetime of cell-cell interactions (t_contact_) 46 was significantly higher in the three N13 clones (1054-1204 min) than in Mock and CS cells (567 min and 676 min, respectively) (Figure 7B). These results indicate that PTPN13 expression affects the phosphorylation status and/or expression of cell-cell adhesion proteins, and consequently regulates cell-cell adhesion dynamics by promoting and stabilizing cell junctions.

As cell junction destabilization is an EMT hallmark, we measured by RT-qPCR the expression of four transcription factors (SLUG, SNAIL, ZEB-1 and ZEB-2) that drive EMT, and of E-cadherin and vimentin (epithelial and mesenchymal marker, respectively). Among the four transcription factors, only *SNAIL* expression, which usually decreases with MET, was possibly increased in N13 and CS cells, but the important variation amongst experiments did not allow reaching a firm conclusion (Figure 7C). E-cadherin expression was very weak in all samples with heterogeneity among clones expressing PTPN13 (Figure 7C). In contrast, in accordance with MET-like phenotype, high vimentin expression was weakly, but significantly decreased (10 to 20%) in N13 clones (Figure 7C). This indicates that PTPN13 is not triggering a conventional MET transition via changes in ZEB, SNAIL or SLUG expression.

To analyze PTPN13 effect on specific cells junctions, we focused on desmoplakin, desmoglein (desmosomes) and TJP-1 (occluding junctions), on the basis of our phosphoproteomic data. Western blot analysis indicated that TJP-1 expression was below detection in all MDA-MB-231 clones (not shown). Conversely, desmoplakin was strongly expressed in the three N13 clones, weakly in CS cells and virtually absent in Mock cells (Figure 7D). This suggests that the increase in the amount of desmoplakin obtained in the phosphoproteomics experiment may be due to an increase in desmoplakin protein levels rather than an increase in the proportion of desmoplakin being phosphorylated. Indeed, immunoprecipitation of phosphotyrosine-containing proteins followed by immunoblot with anti-desmoplakin antibody showed an equivalent proportion of phosphorylated desmoplakin in the N13-2 and CS clones (figure S5A). This result is only partly explained by desmoplakin mRNA level upregulation in N13 (5-8 fold) and CS (3-fold) cells compared with Mock cells (Figure 7E). This suggests that PTPN13 promotes desmoplakin protein stability. Stability experiments using cycloheximide revealed a long half-life of desmoplakin in clone N13-2 (figure S5B), in support of this hypothesis.

**Figure 8:**
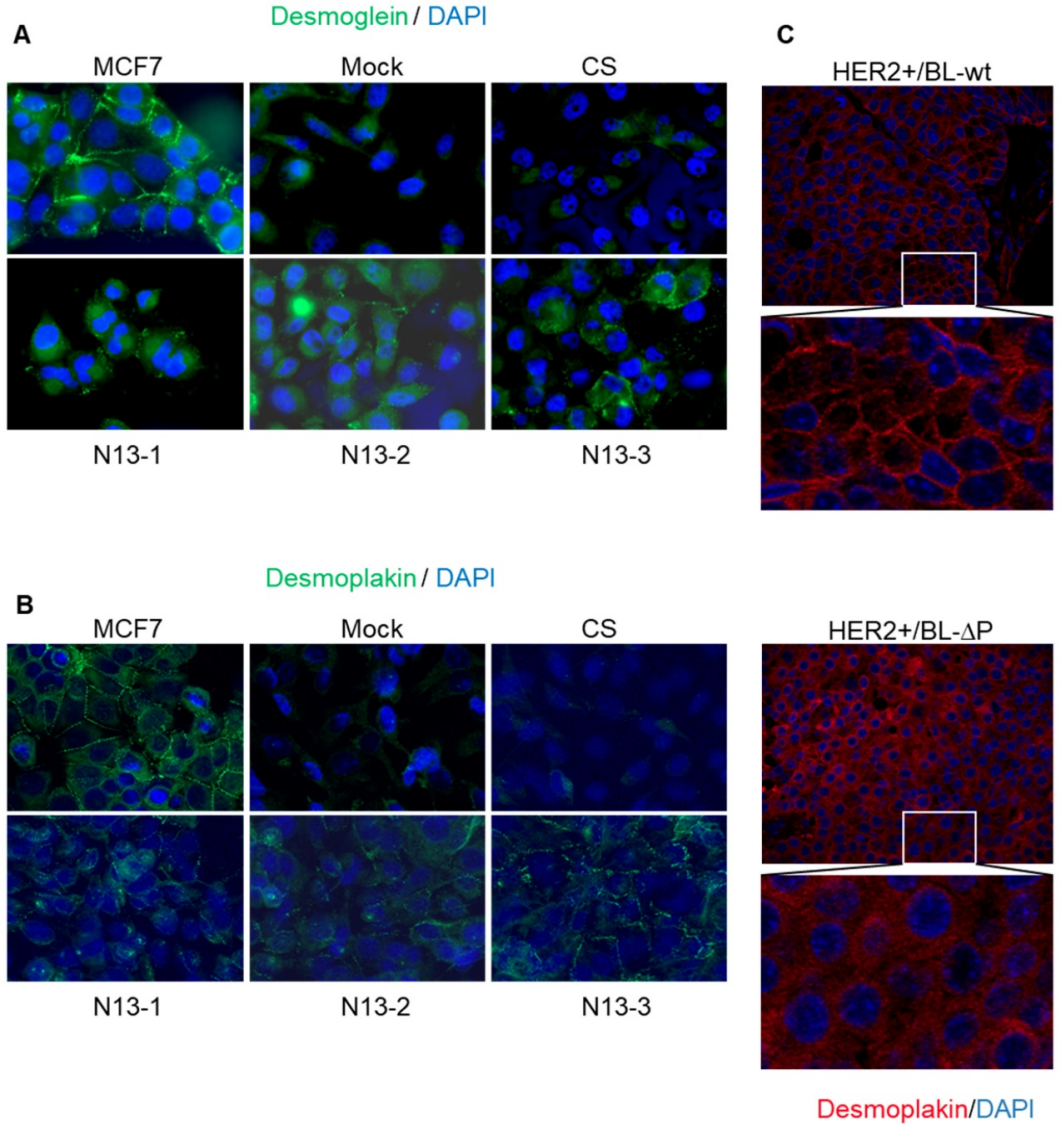
** PTPN13 stabilizes desmosomes *in vitro* and *in vivo*. A&B**: Desmosomes were visualized in the indicated cell clones by immunofluorescence analysis using anti-desmoglein 2 (A) and anti-desmoplakin I+II (B) antibodies; nuclei were counterstained with Hoechst (magnification: x63). **C:** Desmosomes were visualized in tumors from HER2+/BL-wt (upper panels) and HER2+/BL-ΔP (lower panels) mice by immunofluorescence analysis using anti-desmoplakin I+II antibodies; nuclei were counterstained with Hoechst (magnification: x20).

Unlike MCF7 cells, parental MDA-MB-231 cells do not form cell-cell junctions and do not express TJP-1 or desmoglein and desmoplakin at cell-cell contacts (Figure 8A,B). Indeed, we confirmed the absence of visible junctions or desmoplakin staining (Figure 8B) and observed a diffuse desmoglein signal in Mock and CS MDA-MB-231 cells (Figure 8A). In contrast, desmoglein was relocated to cell-cell contacts in N13 cells that also showed a punctate desmoplakin staining at contact sites (Figure 8A,B). These results indicate than PTPN13 positively regulates desmosome assembly and/or stability in MDA-MB-231 cells.

To generalize this finding, we examined whether the presence of EMT patterns at the tumor periphery in HER2+/BL-ΔP mice was associated with cell-cell junction deregulation in these tumors. Comparison of desmoplakin staining in HER2+/BL-ΔP and HER2+/BL-wt tumor specimens highlighted the presence of visible junctions with a punctate desmoplakin staining at cell-cell contacts in 87.5% of HER2+/BL-wt tumors. Conversely, desmoplakin staining intensity was heterogeneous and was located at cell-cell contacts only in 12.5% of HER2+/BL-ΔP tumors (Figure 8C). On the other hand, desmoplakin protein levels were only slightly decreased in mammary organoid cells from PTPBL-ΔP mice as compared to wild type controls (Figure S6). This indicates that the PTPN13 effect on desmoplakin levels is only observed in models with invasive potential, suggesting an indirect protective effect of PTPN13 on desmoplakin by inhibiting pathways that destabilize intercellular junctions.

Altogether, these results indicate than PTPN13 catalytic activity positively regulates desmosome assembly and/or stability in breast cancer cells.

## Discussion

Although previous clinical studies on PTPN13 expression in various tumor types argued for a tumor suppressor role, its exact role in tumorigenesis remains unclear due to its negative contribution to FAS receptor-induced apoptosis 8,26. Here, we used a genetic approach, by crossing mice that lack PTPN13 catalytic activity 9 with transgenic mice that overexpress human HER2 and are more prone to breast cancer 32. We found that genetic deletion of the PTPN13 catalytic domain results in an increased HER2-induced breast tumor development and a more invasive phenotype of resulting tumors. To our knowledge, these results are the first genetic evidence that PTPN13 regulates breast tumor development and aggressiveness. Then, using gain-of-function isogenic clones of MDA-MB-231 (HER2-negative and hormone-independent) breast cancer cells we demonstrated that PTPN13 phosphatase activity inhibits invasion and promotes intercellular adhesion. Intriguingly, this MET-like transition induced by PTPN13 expression did not coincide with modified EMT driver transcription factor levels but was rather associated with increased desmoplakin levels and desmoglein 2 localization at cell-cell contacts.

Using* in vitro* and xenograft models, we previously established that PTPN13 modulates the aggressiveness of the poorly tumorigenic, hormone-dependent MCF7 breast cancer cell line 13. Specifically, in athymic mice, PTPN13 knock-down promoted tumor growth and invasiveness and reduced cell adhesion 13. We now extended our knowledge about PTPN13 effect on cell invasiveness to hormone-independent breast cancer models (i.e., HER2-positive and TNBC tumors). Indeed, in HER2+/ BL-ΔP mice, tumor latency was reduced, whereas breast cancer frequency was increased and tumors showed a more aggressive phenotype (higher mitotic index, multilobular appearance, cancer cell embolization in vessels, and EMT characteristics). In MDA-MB-231 cells, we found that PTPN13 inhibited cell migration and invasion and that its enzymatic activity is required. These findings are in agreement with data obtained using other tumor types. Indeed, PTPN13 inhibits vascular endothelial cell movement through interaction with NECL-4 and VEGFR 47, reduces the invasive capacity of PC3 prostate cancer cells 48, negatively regulates the growth of NCI-H292 lung cancer cells *in vitro* and *in vivo* 20, and inhibits invasion by HEPG2 and 97H hepatocellular carcinoma cell lines 24. Altogether, these results establish PTPN13 inhibitory role in cell migration and invasion in numerous tumor cell models.

Currently, only few PTPN13 substrates have been identified 26 and the signaling pathways controlled by the phosphatase largely remain to be unveiled. We used a SILAC phosphoproteomic approach to compare protein tyrosine phosphorylation in MDA-MB-231 clones that overexpress wt or catalytically inactive PTPN13. We confirmed that IRS1 is a PTPN13 phosphoprotein substrate 30. The PTPN13 substrate Src 13, however, was not detected and we corroborated the absence of Src phosphorylation in our cell culture conditions on Western blot. Gene ontology enrichment analysis clearly showed that differentially phosphorylated proteins were involved mainly in intercellular junction functions. The stability of intercellular junctions plays a crucial protective role against EMT and cancer invasion 49, and is highly regulated by tyrosine phosphorylation 50. Using cell migration and aggregation assays, we confirmed that PTPN13 activity increases and stabilizes cell-cell adhesion in MDA-MB-231 cells. Destabilization of cell junctions is a EMT hallmark and is mediated by expression of SNAIL and ZEB that repress epithelial markers, such as E-cadherin or desmoplakin, and activate the expression of genes associated with a mesenchymal phenotype, such as vimentin 51. We found that PTPN13 expression slightly decreased vimentin and strongly increased desmoplakin expression, reminiscent of a MET. Intriguingly, EMT driver transcription factors were unaffected, with only a slight upregulation of SNAIL1, which is known to repress desmoplakin expression 51. Moreover, PTPN13 expression promoted desmosome formation in MDA-MB-231 cells and in HER2+/BL-wt breast tumors, as revealed by desmoglein and desmoplakin immunostaining. In addition, our data suggest a positive effect of PTPN13 activity on desmoplakin protein levels. Knowing that the general stabilization of the junctions stabilizes desmoplakin 52, the observed effect may be indirect through the stabilization of other junction components. In line, our phosphoproteomic study points to enrichment of not only desmosomes but also of more general cell junctional structures and occluding junctions. While the association between desmosome stability and invasion is less well established than for adherens junctions, the use of genetic mouse models has shown that desmoplakin deletion promotes tumor microinvasion in pancreatic neuroendocrine carcinogenesis 53, and that loss of the desmosomal protein PERP stimulates skin cancer development and progression 54. Furthermore, desmoglein 2 silencing leads to loss of cell cohesion and increases migration and invasion of pancreatic adenocarcinoma cells 55.

We previously established that PTPN13 mRNA expression level is an independent prognostic indicator of favorable outcome for patients with breast cancer 23, and that PTPN13 protein levels are decreased in primary and metastatic breast cancer compared with normal breast tissue 13. We now used KM Plotter data 36 to analyze the overall survival of patients with breast cancer according to the tumor PTPN13 RNA level (high/low), and confirmed the prognostic interest of PTPN13. These results are corroborated by other studies. Wei and collaborators found that a nucleotide polymorphisms in PTPN13 that is associated with colorectal cancer susceptibility also influences the risk of breast cancer in a Chinese Han population 56. Furthermore, studies in ovarian 57, prostate 48 and hepatocellular carcinoma 24 showed loss of PTPN13 expression in tumor versus normal tissues, in high grade versus low grade tumors, and a correlation between PTPN13 expression and favorable prognosis, respectively. Similarly, a bio-statistical analysis identified PTPN13 as a hub gene in a lung cancer network and demonstrated its prognostic importance in four independent lung cancer datasets 58. We have shown that the PTP13 inhibition of breast cancers is associated with a stabilization of cell-junctions; it would now be important to investigate whether such PTPN13 effects are found in all these tumor types where PTPN13 expression is associated with a good prognosis.

In summary, using genetically modified mouse and cell models we demonstrated that PTPN13 acts as a tumor suppressor in breast cancer and that it inhibits tumor aggressiveness through a SNAIL- and ZEB-independent MET-like transition, cautioning against the use of PTPN13 as a therapeutic target to increase apoptosis during cancer therapies 59,60. Additional studies are now required to determine how PTPN13 catalytic activity is regulated and its precise role in cell-cell junctions. On one hand, PTPN13 dephosphorylates signal transducers and activators of transcription (61) that are able to regulate EMT in ovarian cancers (62). On the other hand, PTPN13 is involved in proteostasis-regulating processes (e.g., proteosomal degradation 63 and autophagy 64) that are crucial determinants of cell-matrix 65 and cell-cell 66,67 interactions and consequently can affect tumor cell dissemination. In addition, recent results show close relationships between PDZ-domain proteins, cell junctions and the regulation of Fas-induced apoptosis 68. Taken together, our findings strengthen the interest to unravel the signaling pathways regulated by PTPN13 to identify new therapeutic targets involved in cell tumor invasion and metastasis formation.

## Supplementary Material

Supplementary methods and figures.

Table S1.

Table S2.

## Abbreviations

FDR: false discovery rate
FRT: Flp recombination target
IRS1: insulin receptor substrate 1
MET: mesenchymal-epithelial transition
PTK: protein tyrosine kinases
PTPs: protein tyrosine phosphatases
qPCR: quantitative polymerase chain reactions
TNBC: triple-negative breast cancer
