# Supplementary material for: PTPN13 induces cell junction stabilization and inhibits mammary tumor invasiveness
Source: Theranostics. 2020 Jan 1;10(3):1016–32. doi: 10.7150/thno.38537 (PMC6956795; doi:10.7150/thno.38537)
Supplement: Supplementary file 1 — Supplementary methods and figures. [file thnov10p1016s1.pdf]

Supplementary Methods

Proteomics

Cell culture and sample preparation

For SILAC experiments, a modified method previously described was used (59). Briefly, two opposite biological replicates were performed simultaneously by switching the culture conditions and the cell lines (N13-2 and CS clones). Cells were grown either in heavy or light medium. SILAC media composition: the appropriate amounts of heavy L-arginine [13C6,15N4] and heavy L-lysine [13C6,15N2] (Cambridge Isotope Laboratories) or light L-arginine [12C6,14N4] and light L-lysine [12C6,14N2] (Sigma, Saint Quentin Fallavier, France) were added to lysine- and arginine-free DMEM (Euromedex, Souffelweyersheim, France) supplemented with 10% dialyzed FBS (Invitrogen Corp.) and L-methionine (100 mg/ml; Sigma, Saint Quentin Fallavier, France).

After 11 days of cell culture, cells were serum-starved overnight and then stimulated by switching to complete medium for 30min. The isotope integration rate reached 95%. Cells were lysed as described in the Western blot analysis section. After overnight incubation with anti-phosphotyrosine immunoaffinity beads PY99 (Santa Cruz Biotechnology) at 4°C under rotation, phosphoproteins were then subjected to DTT reduction and IAA alkylation, and size-separated on 12% SDS-PAGE gels. Proteins were in-gel digested with trypsin (GOLD Promega 1 µg/µl in 50mM acetic acid). The resulting peptides were extracted by series of acetonitrile dehydration/triethylammonium bicarbonate buffer (1M) rehydration and vacuum-dried.

Mass spectrometric analysis

Samples (1 µl) were analyzed online using a nonoESI Qexactive mass spectrometer (Thermo Fisher Scientific, Waltham, MA) coupled with a RSLC HPLC (Dionex,

Amsterdam, The Netherlands). Sample desalting and pre-concentration were performed on-line on a Pepmap® precolumn (0.3 mm x 10 mm). A gradient consisting of 2-24% B for 68min, 24-40% B for 15min, 40-72% B for 5min, and 80% B for 10min (A = 0.1% formic acid, 2% acetonitrile in water; B = 0.1 % formic acid in acetonitrile) at 300 nl/min was used to elute peptides from the capillary (0.075 mm x 150 mm) reverse-phase column (Pepmap®, Dionex). Nano-ESI was performed with a spray voltage of 1.9 kV and a heated capillary temperature of 250°C. Cycles of one full-scan mass spectrum (350–1500 m/z) at a resolution of 70,000, followed by ten data-dependent MS/MS spectra were repeated continuously throughout the nanoLC separation. All MS/MS spectra were recorded at a resolution of 17,500 with an isolation window of 2 m/z (AGC target 1e5, NCE 26). Data were acquired using the Xcalibur software (v 2.2, Thermo Fisher Scientific, Waltham, MA).

Raw data were analyzed using the MaxQuant software (V. 1.4.1.2). Retention time-dependent mass recalibration was applied with the aid of a first search as implemented in the Andromeda software, and peak lists were searched against the UniProt human database (Complete proteome set with isoform; http://www.uniprot.org), 255 frequently observed contaminants as well as reversed sequences of all entries. The standard MaxQuant settings were used. Enzyme specificity was set to Trypsin/P. Up to two missed cleavages were allowed and only peptides with at least seven amino acids in length were considered. Methionine oxidation and serine, threonine and tyrosine phosphorylation were set as variable modifications. Peptide identifications were accepted based on their false discovery rate (FDR, 1%). Accepted peptide sequences were subsequently assembled by MaxQuant into proteins, to achieve a false discovery rate of 1% at the protein level. Relative protein quantification in samples to be compared

was performed based on the median SILAC ratios, using the MaxQuant software with standard settings.

Statistical analysis

Proteins quantified in both biological replicates with at least a total number of three peptide evidences were selected. To analyze the normalized SILAC ratios of all peptide evidences of each protein, an empirical estimation of their FDR was performed based on the non-parametric Wilcoxon rank test. Proteins were accepted as differentially enriched based on their FDR (0.05%) and their SILAC ratio (>20% of variation in both biological replicates).

Improving the identification of enriched proteins in SILAC data

Although the SILAC approach is now frequently used, there are still no dedicated tools allowing an exhaustive analysis of the data and there is no consensus on straightforward statistical tests to identify proteins of which the phosphorylation is significantly changed, based on their SILAC ratios. The difficulty in interpreting the values of these ratios is related to the fact that: (i) there are few replicates (often only two), (ii) there is a wide diversity in the number of peptides identified per protein and (iii) there may be errors in assigning peptides to particular proteins. Furthermore, in quantitative phosphoproteomics one has to combine peptide-level values with protein-level values and perform tests at each level. The correspondence can be achieved in several ways and classical statistical methods designed for microarrays (simple Student's *t*-test, LIMMA) do not cover this more complex situation or need a lot of replicates. We therefore designed our own bioinformatics pipeline to go through most of the analysis steps, including statistical tests. For the first step of the SILAC analysis, which is associating peptides to proteins and remove contaminants, we relied on the existing MaxQuant software. Then, our pipeline merges experiments reversing some ratios depending on the experimental protocol. Only proteins with at least 3 peptide evidences are selected. The next steps combine peptide-level

values to obtain protein scores. We have used the non-parametric Wilcoxon rank test to analyse peptide counts. The p-values obtained for proteins with different number of peptides cannot be compared. To overcome this limitation, we performed false discovery rate (FDR) estimations by comparing the p-value obtained for each protein, with these obtained in thousands of random permutations. The resulting scores can be used to rank all proteins.

Fig S1

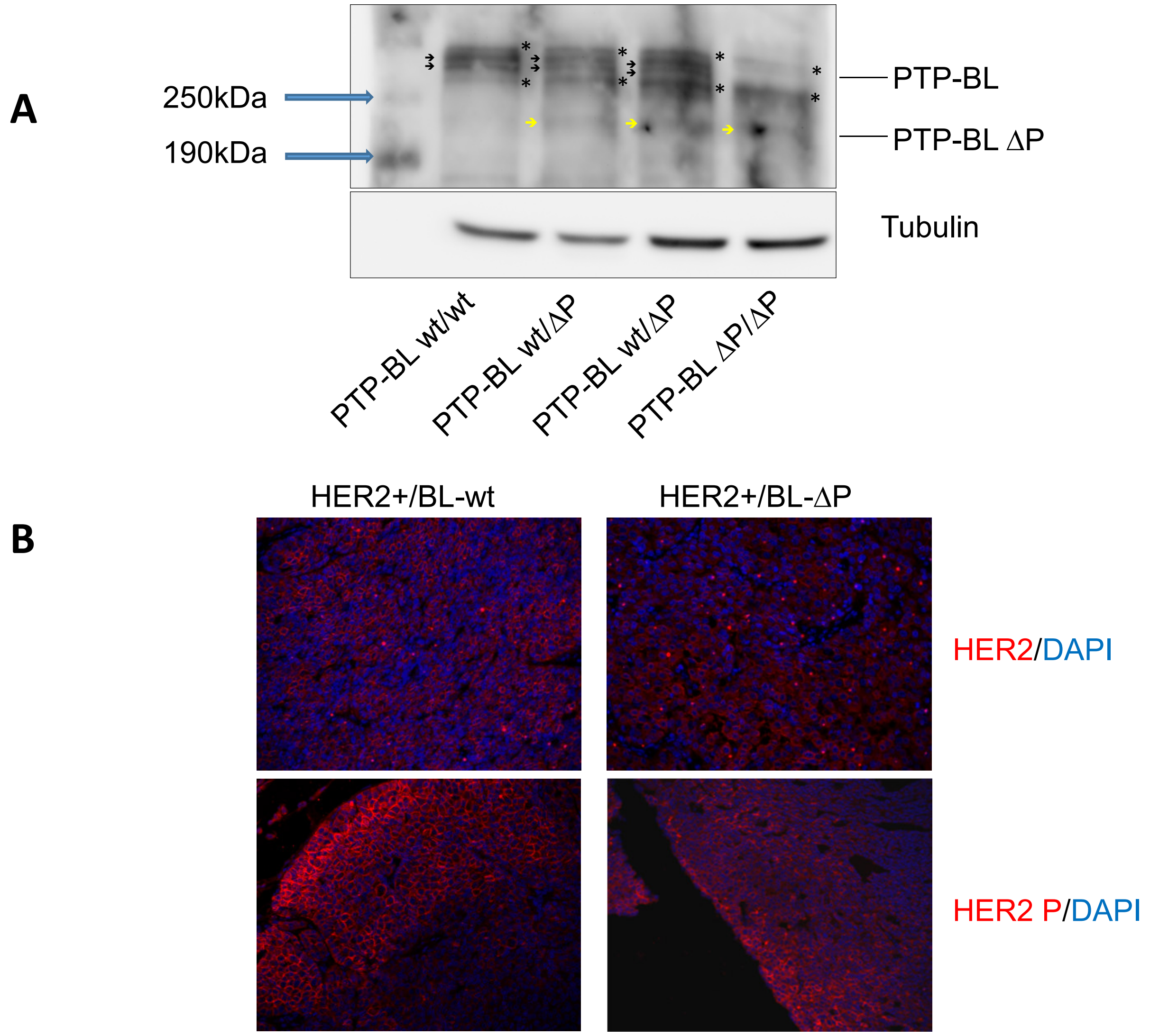

Figure S1 : A: PTP-BL expression in mammary gland from PTP-BL wt/wt and PTP-BL ΔP/ΔP mice. PTP-BL expression in indicated normal primary mammary organoids was monitored by western blotting using BL-N rabbit antiserum (9), with tubulin as loading control. Primary mammary organoids are prepared as described in Ewald AJ, Brenot A, Duong M, Chan BS, Werb Z. Dev Cell. 2008 Apr;14(4):570-81. Two WT PTP-BL isoforms, probably different splice isoforms (white arrows), can be detected in samples of WT and heterozygous mice. Truncated PTP-BL ΔP (yellow arrows) can be detected in organoids of PTP-BL ΔP/ΔP and heterozygous mice, Asterisks indicate nonspecific signals, unrelated to PTP-BL, observed in all sample (as observed by Wansink et al (9) Figure 3).

B: HER2 Phosphorylation on tumors from HER2+/BL-wt and HER2+/BL-ΔP mice. HER2 and HER2 Phosphorylation was visualized in tumors from HER2+/BL-wt (left panels) and HER2+/BL-ΔP (right panels) mice by immunofluorescence analysis using anti-HER2 (from cell signaling technology ref 2242, upper panel) and anti-HER2 Ptyr1221/1222 (from cell signaling technology ref2243 lower panel) ; nuclei were counterstained with Hoechst (magnification: x20).

Fig S2

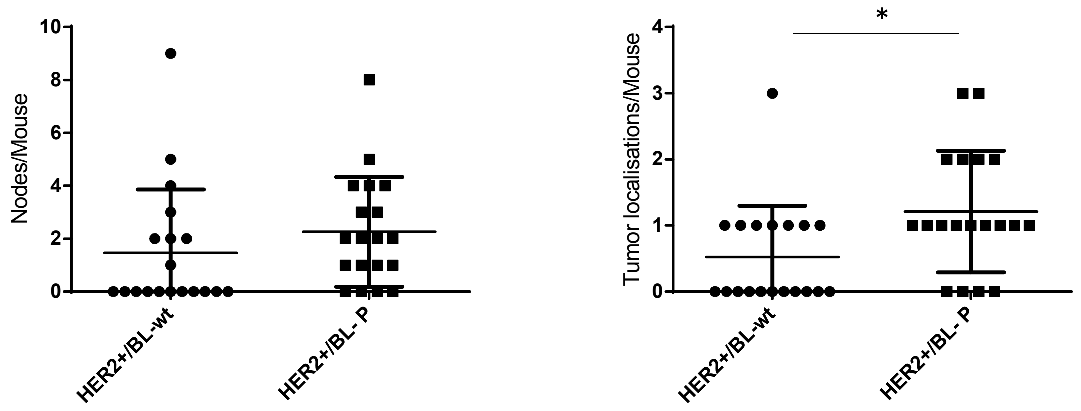

Figure S2: Lack of catalytically active PTPN13 promotes number of tumor localization per mouse. Number of nodule and tumors per mouse was represented with mean and SD. *P<0.05 (two-tailed Mann-Whitney test).

Fig S3

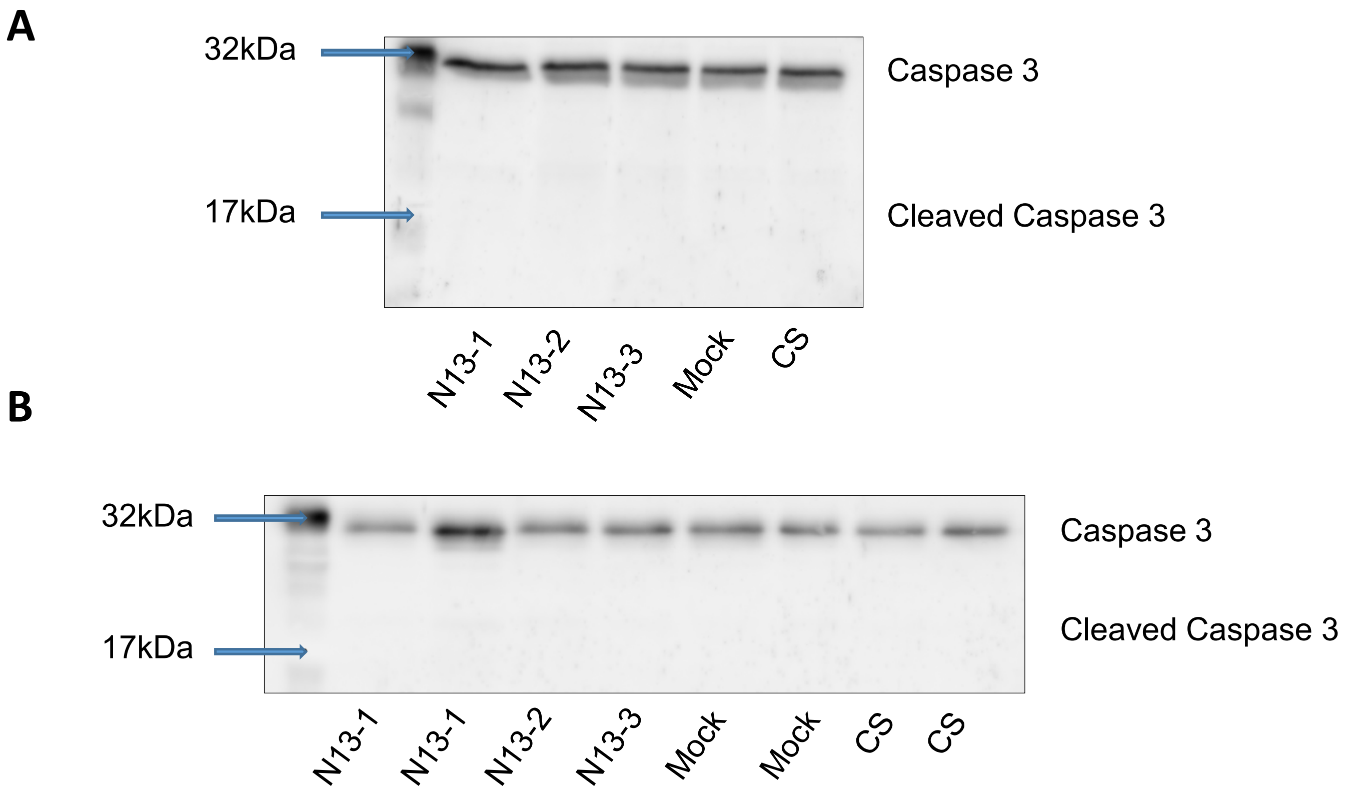

Figure S3 : Absence of caspase 3 cleavage. **A:** Caspase3 and cleaved caspase 3 expression in the indicated cell clones was monitored by western blotting on total cell lysate using anti caspase 3 antibody (Cell signaling technology ref 9362). **B:** Caspase3 and cleaved caspase 3 expression in the indicated xenograft was monitored by western blotting on total lysate using anti caspase 3 antibody (Cell signaling technology ref 9362). Native caspase 3 (27-30 kDa) was detected on all sample, cleaved caspase 3 (15-19kDa) was undetectable.

Fig S4

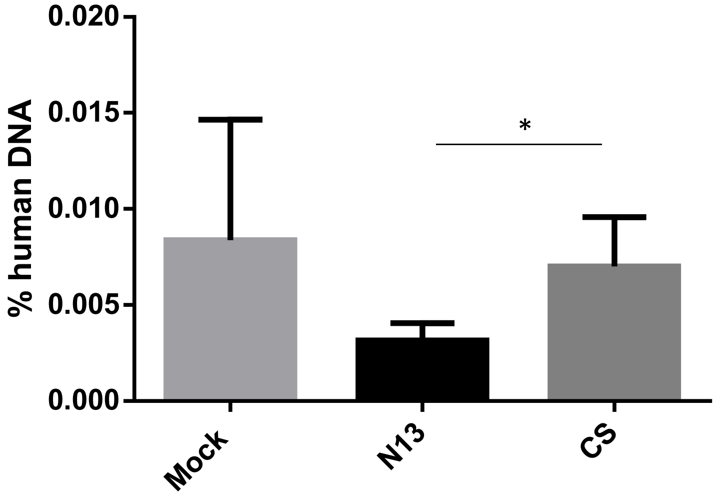

Figure S4 : Micro-metastasis detection by PCR. Data represent mean +/- SEM of the percentage of human DNA in lung or liver from 37 (Mock) or 12 (N13 and CS) murine tissues. *P<0.05 (two-tailed Mann-Whitney test). The percentage of human DNA in murine tissues was calculated as indicated in the materials and methods according to the method written by Becker et al (38).

Fig S5

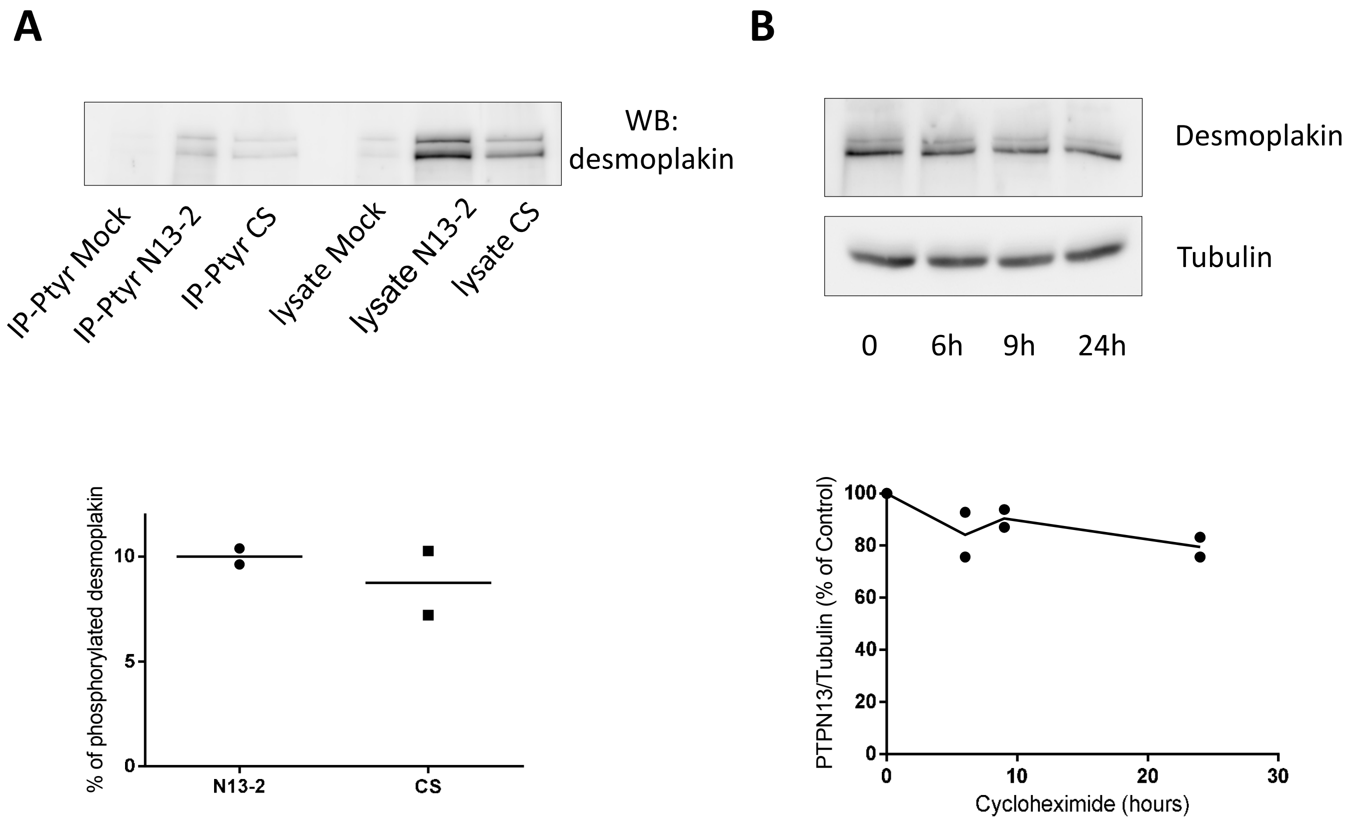

Figure S5 :Desmoplakin phosphorylation and half-life. A: Upper panel. Desmoplakin expression in the indicated cell clones was monitored by western blotting on total cell lysate and its phosphorylation by western blotting after immunoprecipitation with PY99 antibody (Santa Cruz biotechnology ref sc-7030). **Lower panel:** quantification of two independent experiments. **B: Upper panel.** Desmoplakin expression in N13-2 clone was monitored by western blotting with tubulin as loading control on total cell lysate after indicated time of cycloheximide treatment (25µg/ml). **Lower panel:** quantification of two independent experiments.

Fig S6

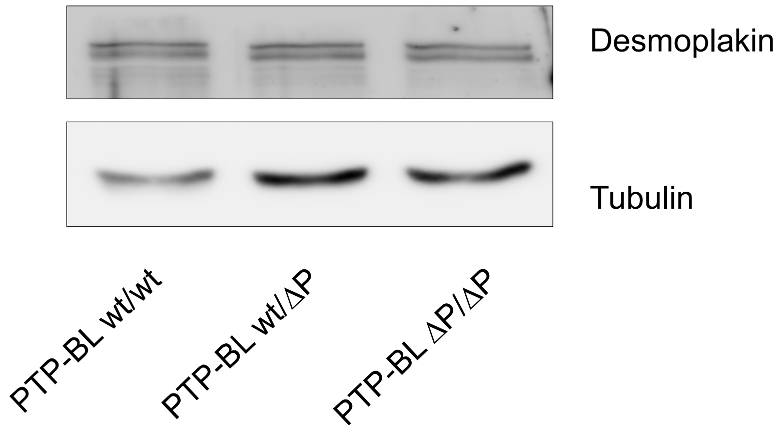

Figure S6: Desmoplakin expression in mammary gland from PTP-BL wt/wt and PTP-BL ΔP/ΔP mice. Desmoplakin expression in normal primary mammary organoids was monitored by western blotting, with tubulin as loading control. Primary mammary organoids are prepared as described in Ewald AJ, Brenot A, Duong M, Chan BS, Werb Z. Collective epithelial migration and cell rearrangements drive mammary branching morphogenesis. Dev Cell. 2008 Apr;14(4):570-81.
